# Naphth[1,2-*d*]imidazoles Bioactive from β-Lapachone: Fluorescent Probes and Cytotoxic Agents to Cancer Cells

**DOI:** 10.3390/molecules28073008

**Published:** 2023-03-28

**Authors:** Victória Laysna dos Anjos Santos, Arlan de Assis Gonsalves, Délis Galvão Guimarães, Sidney Silva Simplicio, Helinando Pequeno de Oliveira, Lara Polyana Silva Ramos, Marcília Pinheiro da Costa, Fátima de Cássia Evangelista de Oliveira, Claudia Pessoa, Cleônia Roberta Melo Araújo

**Affiliations:** 1 Graduate Program in Health and Biological Sciences, Universidade Federal do Vale do São Francisco, Av. José de Sá Maniçoba s/n, Campus Centro, Petrolina 56304-917, Brazil; victoria.laysna@univasf.edu.br (V.L.d.A.S.); arlan.gonsalves@univasf.edu.br (A.d.A.G.); sidney.simplicio@discente.univasf.edu.br (S.S.S.); 2 Graduate Program in Materials Science, Universidade Federal do Vale do São Francisco, Av. Antônio Carlos Magalhães 510, Campus Juazeiro, Juazeiro 48902-300, Brazil; helinando.oliveira@univasf.edu.br; 3 Graduate Program in Biotechnology, Rede Nordeste de Biotecnologia—RENORBIO, Centro de Biociências, Universidade Federal do Rio Grande do Norte, Campus Lagoa Nova, Natal 59078-900, Brazil; delisgalvao@gmail.com; 4 Graduate Program in Pharmaceutical Sciences, Universidade Federal do Piauí, Teresina 64049-550, Brazil; lara_poliana@hotmail.com (L.P.S.R.);; 5 Graduate Program in Physiology and Pharmacology, Universidade Federal do Ceará, Fortaleza 60430-270, Brazil; cassiadefatima3006@gmail.com (F.d.C.E.d.O.); cpessoa@ufc.br (C.P.)

**Keywords:** heterocycle, anticancer, fluorescent probe, theranostic, naphthoimidazole

## Abstract

Theranostics combines therapeutic and imaging diagnostic techniques that are extremely dependent on the action of imaging agent, transporter of therapeutic molecules, and specific target ligand, in which fluorescent probes can act as diagnostic agents. In particular, naphthoimidazoles are potential bioactive heterocycle compounds to be used in several biomedical applications. With this aim, a group of seven naphth[1,2-*d*]imidazole compounds were synthesized from β-lapachone. Their optical properties and their cytotoxic activity against cancer cells and their compounds were evaluated and confirmed promising values for molar absorptivity coefficients (on the order of 10^3^ to 10^4^), intense fluorescence emissions in the blue region, and large Stokes shifts (20–103 nm). Furthermore, the probes were also selective for analyzed cancer cells (leukemic cells (HL-60). The naphth[1,2-*d*]imidazoles showed IC_50_ between 8.71 and 29.92 μM against HL-60 cells. For HCT-116 cells, values for IC_50_ between 21.12 and 62.11 μM were observed. The selective cytotoxicity towards cancer cells and the fluorescence of the synthesized naphth[1,2-*d*]imidazoles are promising responses that make possible the application of these components in antitumor theranostic systems.

## 1. Introduction

The multifunctionality of theranostic agents introduces several advantages for medicine, overcoming pharmacokinetic and selectivity issues of conventional therapy and diagnostic agents [1], while providing the image monitoring of pathology progression as well as the pharmacokinetic profile of the drug in the body [2].

The design of a theranostic agent requires a combination of different areas, such as chemistry, physics, nanotechnology, biochemistry, and engineering, with the aim of obtaining a multifunctional platform capable of performing non-invasive therapy and diagnosis of a pathological condition [3]. Typically, a theranostic agent is composed of (i) an imaging agent, (ii) a therapeutic molecule, (iii) a target-specific ligand, and (iv) a carrier. The diagnostic agent is a fundamental part of a theranostic system. It favors the non-invasive visualization of cellular and subcellular processes of a pathological condition through image emission. Examples of these components include fluorophores with the ability to respond to specific stimuli regarding the identification of biological species [4,5].

Fluorescent compounds, such as naphthoxazoles, have been explored as active molecules in biological systems [6]. Imidazoles and oxazoles can be synthesized through a multicomponent reaction, the Debus–Radziszewski reaction, employing α-dicarbonyl compounds and aldehydes [7,8]. The same reaction generates naphthoimidazoles and naphthoxazoles in which 1,2-naphthoquinones are used as α-dicarbonyl compounds [9,10]. β-Lapachone is a 1,2-naphthoquinone originally isolated from the heartwood of *Handroanthus impetiginosus* [11], which can be considered a potential antitumor agent [12,13,14]. The efficacy of β-lapachone for cancer treatment was evaluated through phase I and II clinical trials, with the naphthoquinone in the form of ARQ 501 and ARQ 761 [15,16,17,18]. However, several drawbacks of β-lapachone, such as low water solubility and narrow therapeutic windows, limited its clinical applications [19,20].

**Figure 1:**
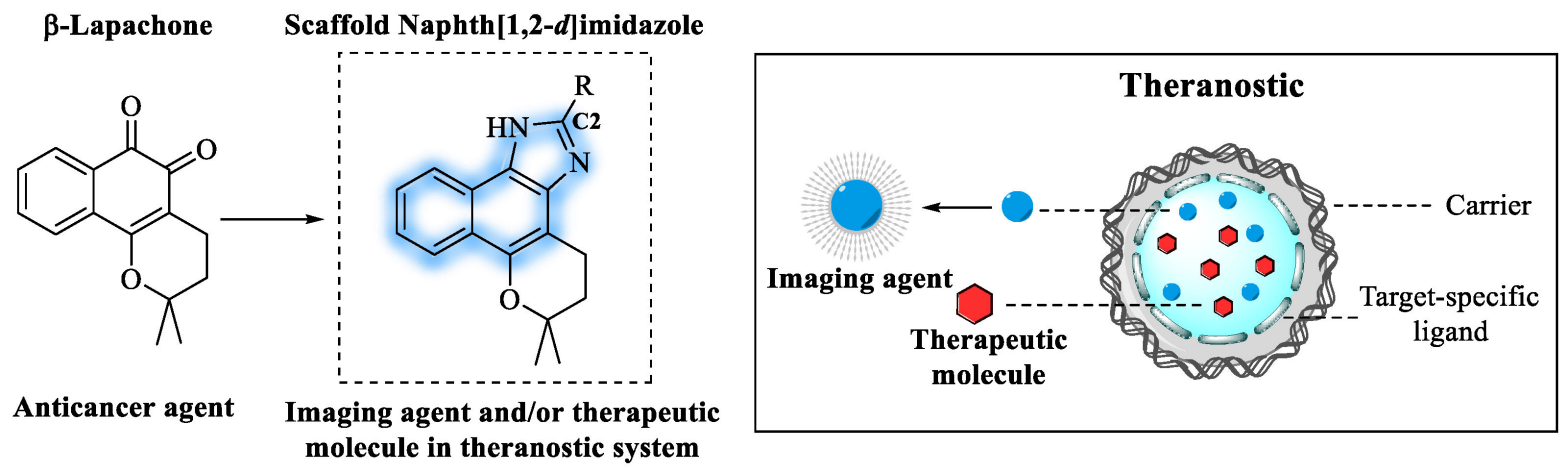
Molecular design of the naphth[1,2-*d*]imidazoles with anticancer and fluorescent properties.

Based on the cytotoxicity of β-lapachone and the fluorescent properties of naphthoazole heterocycles, the scaffold 1,2-naphtho[1,2-*d*]imidazole was designed to be a fluorescent emitter with antitumor action, being considered a promising component of theranostic systems, as shown in Figure 1.

Herein, it is reported the synthesis of different naphth[1,2-*d*]imidazoles, with modification of substituents at the C2 position of the naphthoimidazole ring, and the following evaluation of their photophysical and anticancer activity as a part of a strategy to provide a new class of materials with promising biomedical applicability.

## 2. Results and Discussion

**Scheme 1:**
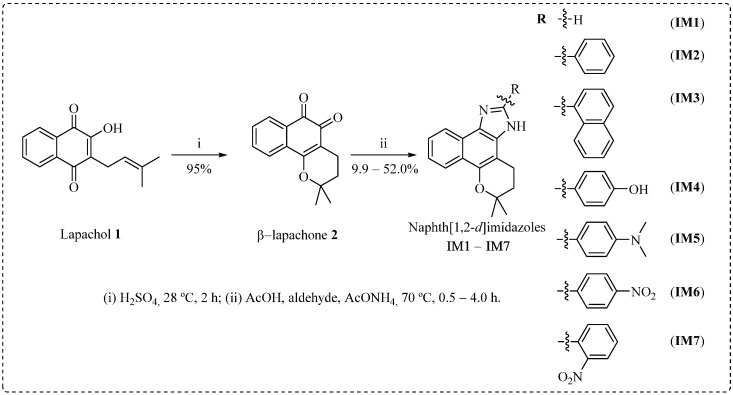
Synthetic route to obtain the naphth[1,2-*d*]imidazoles **IM1**–**IM7**.

Naphth[1,2-*d*]imidazoles **IM1**–**IM7** were prepared in two steps (Scheme 1) from lapachol **1**, in which the natural 1,4-naphthoquinone was extracted from the heartwood of trees of the genus *Tabebuia*. In the first step, β-lapachone (β-Lap **2**) was obtained from the acid-catalyzed cyclization of lapachol **1** using sulfuric acid (H_2_SO_4_). In the following step, the compounds **IM1**–**IM7** were synthesized through the Debus–Radziszewski reaction, in a one-pot process between β-Lap **2** and the corresponding aldehyde, using ammonium acetate as a source of ammonia (Scheme 1). The reactions were established at 70 °C in acetic acid with a reaction time range of 0.5–4.0 h. The crude reactions were treated with sodium bisulfite (NaHSO_3_), and the products were purified using column chromatography or recrystallization. The naphthoimidazoles returned yields in the range of 9.9 to 52.0%, and their structures were scrutinized by analyzing the 1D and 2D Nuclear Magnetic Resonance (NMR) spectra, mass spectroscopy, and Fourier Transform Infrared (FTIR).

### 2.1. Optical Properties—Studies of Absorption and Fluorescence Spectra

Fluorescent molecules with the ability to absorb ultraviolet radiation and to emit in a range of wavelengths greater than that absorbed, that is, in the visible region, are extremely important to biomedical applications [21]. The characteristic time for the fluorescence process (on the order of 10^−9^ s) depends on the interaction of molecules with the surrounding environment, being attractive for the evaluation of several phenomena from the biophysical properties of molecules [21]. Thus, the photophysical study of the naphth[1,2-*d*]imidazoles **IM1**–**IM7** synthesized was carried out to verify their potential to emission of molecules for potential use in theranostic systems. The data for Ultraviolet–visible (UV–vis) absorption and fluorescence spectroscopy from naphth[1,2-*d*]imidazoles **IM1**–**IM7** are summarized in Table 1.

#### 2.1.1. Solvatochromism Study

The dispersed molecules in a specific solvent can interact with other molecules of a fluorophore, affecting their emissive properties [22]. This phenomenon is called solvatochromism and depends on factors such as the polarity of the solvent, hydrogen bonding ability, pH, and viscosity of the solvent [22,23]. Considering the application of fluorescent probes in living cells and tissues, the solvatochromism study can evaluate the sensitivity and selectivity of the new compounds [24].

The influence of the solvent on optical characteristics of the synthesized naphth[1,2-*d*]imidazoles **IM1**–**IM7** was evaluated from the solvatochromism study that considered four solvents: hexane, dichloromethane (CH_2_Cl_2_), dimethyl sulfoxide (DMSO), and methanol (CH_3_OH) (Table S1). From the UV–vis absorption spectra of the naphtha[1,2-*d*]imidazoles (for different solvents), it was possible to determine the most suitable solvents and wavelengths for further photophysical studies. The criteria considered solvents for naphth[1,2-*d*]imidazoles that presented positive solvatochromism, i.e., redshift with increasing polarity of the solvent.

The absorption spectra of 2-substituted naphth[1,2-*d*]imidazoles (**IM2**–**IM7**) showed two absorption bands in the ultraviolet region, corresponding to the π→π* transition of the substituent at the carbon C2 of the naphthoimidazole ring (~314 nm) and of the imidazole ring (~363 nm) [25,26,27] (Table S1).

The naphth[1,2-*d*]imidazoles **IM2** and **IM6** showed a higher bathochromic shift for polar solvents, a strong influence of the increasing solvent polarity on the absorption spectrum of these derivatives. On the other hand, **IM7** showed a positive bathochromic effect in comparison to hexane, a nonpolar solvent. The other naphthoimidazoles (**IM1**, **IM3**, **IM4**, and **IM5**) exhibited an increasing redshift in CH_2_Cl_2_ (Table S1).

#### 2.1.2. Molar Absorption Coefficient

The molar absorption coefficient (ε_Abs_) is also an important parameter for the development of fluorescent probes. Samples prepared with naphth[1,2-*d*]imidazoles **IM1**–**IM7** showed high ε_Abs_ on the order of 10^3^ to 10^4^ M^−1^ cm^−1^ (Table 1), which is consistent with the allowed transition π→π* of π-conjugated systems [26,28,29,30] characteristic of the imidazole nucleus [25,31].

**Table 1:** Photophysical properties of the naphth[1,2-*d*]imidazoles **IM1**–**IM7**.

Compound	Solvent	λ_max_ (nm)	ε_Abs_ ^a^ (10^4^ M^−1^cm^−1^)	λ_emis_ ^b^ (nm)	Δ_ST_ ^c^ (nm)
**IM1**	CH_2_Cl_2_	316	0.61	366	50
**IM2**	DMSO	342	2.43	402	60
**IM3**	CH_2_Cl_2_	354	1.57	457	103
**IM4**	CH_2_Cl_2_	344	1.48	393	49
**IM5**	CH_2_Cl_2_	368	2.71	422	54
**IM6**	CH_3_OH	411	1.85	391	20
**IM7**	Hexane	337	1.35	385	48
DAPI	H_2_O	343 ^d^	-	452	112

^a^: Molar absorptivity coefficient at the concentration of 20 μM. ^b^: Emission wavelength after excitation at 345 nm. ^c^: Stokes shift. ^d^: Data obtained from Farahat et al. (2017) [32].

#### 2.1.3. Fluorescence Spectroscopy Experiments

**Figure 2:**
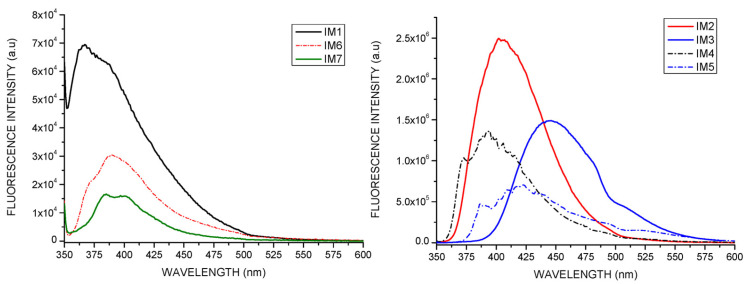
Fluorescence emission spectra of the synthesized naphth[1,2-*d*]imidazoles (λ_Exc_ 345 nm).

Considering the UV–vis absorption spectra of the naphth[1,2-*d*]imidazoles **IM1**–**IM7**, the excitation wavelength of 345 nm was chosen to obtain the fluorescence spectra. Analyzing the fluorescence emission spectra of compounds **IM1**–**IM7**, the emission was observed in the UV–vis region, between the λ_emis_ values of 366 and 457 nm (Figure 2). **IM3** (λ_emis_ 457 nm) and **IM5** (λ_emis_ 422 nm), emitted at lower energy wavelengths, shifted more to the blue region. On the other hand, **IM1**, emitted at a higher energy wavelength, shifted toward the violet region (Figure 2).

In addition, **IM2** (2.49 × 10^6^ au) and **IM3** (1.41 × 10^6^ au) showed fluorescence emission intensities that were thirty-six and twenty-three times greater, respectively, than **IM1** (6.90 × 10^4^ au), indicating that the substitution at the carbon C2 of the naphthoimidazole ring favored the fluorescence emission of naphth[1,2-*d*]imidazoles **IM1**–**IM7** (Figure 2). The increase in the fluorescence evaluated by the substitution of the carbon C2 position is due to the increase in the conjugation of double bonds, providing more-effective intramolecular displacement of electrons [25,33].

By comparison between the fluorescence of **IM4**, **IM5**, and **IM6**, a positive influence on fluorescence was observed in compounds containing electron-donor substituents in the aromatic ring located at C2 of the naphth[1,2-*d*]imidazoles with **IM4** and **IM5** substituted with 4-hydroxyphenyl and 4-dimethylaminophenyl, respectively, showing higher fluorescence intensity than **IM6**, substituted with 4-nitrophenyl. The higher fluorescence of **IM4** and **IM5**, if compared with other fluorophores, is attributed to a possible intramolecular charge transfer (ICT) [34].

Considering the fluorescence intensity emitted, the **IM2**, followed by the **IM3**, **IM4**, and **IM5** compounds, showed the best results. All samples presented higher fluorescence than that observed for the 4′,6-diamidino-2-phenylindole (DAPI), the fluorescent DNA marker [35].

**Figure 3:**
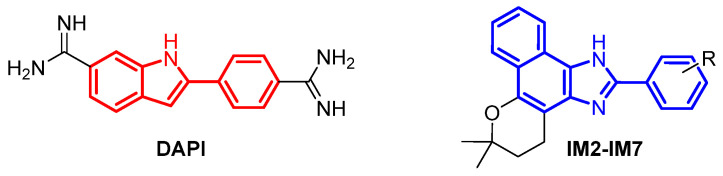
Structural similarity of DAPI and synthesized naphth[1,2-*d*]imidazoles.

DAPI exhibits photophysical characteristics of absorption and emission (λ_abs_ 340 nm and λ_emis_ 453–461 nm) similar to the synthesized naphth[1,2-*d*]imidazoles. By comparison of the fluorescence emission of DAPI (1.13 × 10^5^ au) to that of **IM2** (2.49 × 10^6^ au), **IM3** (1.41 × 10^6^ au), **IM4** (1.36 × 10^6^ au), and **IM5** (7.04 × 10^5^ au), it was observed that IM compounds also present more intense fluorescence in the blue region than DAPI. This may be due to the extension of the conjugated double-bond system of the synthesized naphth[1,2-*d*]imidazoles enhanced by the presence of the naphthalene system associated with the 2-substituted imidazole (Figure 3).

#### 2.1.4. Stokes Shift

Fluorophores with large Stokes shifts (Δ_ST_) are considered promising fluorescent probes for application in vivo cell-imaging studies, since they could minimize the background fluorescence in live tissues [36,37,38,39,40]. The naphth[1,2-*d*]imidazoles **IM1**–**IM7** presented Δ_ST_ between 20 and 103 nm (Table 2). If compared with the structure and Δ_ST_ of naphth[1,2-*d*]imidazoles **IM1**–**IM7**, it was observed that the substituents at C2 affect the ability of this compound to be a fluorophore. **IM1** has no substituents on C2 and was the one with the smallest Δ_ST_, as well as the lowest fluorescence emission (6.90 × 10^4^ au). **IM3** presented the largest displacement, which is substituted at C2 with a naphthyl group. It was also observed that the introduction of electron-withdrawing substituents, such as nitrophenyl, produced naphthoimidazoles with narrow Δ_ST_, as shown for **IM6** (Δ_ST_ 20 nm) and **IM7** (Δ_ST_ 48 nm).

### 2.2. Cytotoxicity Assay

The cytotoxic activity of the naphth[1,2-*d*]imidazoles **IM1**–**IM7** was assessed through colorimetric MTT assay [41,42]. The ability of these compounds to inhibit cell growth against human glioblastoma (SNB-19), human colorectal carcinoma (HCT-116), and human promyelocytic leukemia (HL-60) cell lines was evaluated. The IC_50_ was determined for those compounds, returning a percentage inhibition of cell growth above 75% in at least two tested cell lines. Thus, from all seven naphth[1,2-*d*]imidazoles tested (**IM1**–**IM7**), only **IM3** displayed low growth inhibition against all cell lines and did not have the IC_50_ calculated due to low cytotoxic activity. Doxorubicin was used as the positive control, and cytotoxic activities were expressed as IC_50_ for all the naphth[1,2-*d*]imidazoles in Table 2. The substances that displayed significant results against the cancer cell lines were also investigated against a nontumor cell line of murine fibroblast (L929) to evaluate their selectivity index (SI) (Table 2).

As shown in Table 2, most of the naphth[1,2-*d*]imidazoles are characterized by a certain degree of cytotoxicity against at least one of the malignant cell lines tested. The IC_50_ data showed that **IM1** was the least potent imidazole of the series, demonstrating the importance of the substitution at the C2 carbon of the naphth[1,2-*d*]imidazole ring for the cytotoxic activity against the tested cancer cell lines. If considering the comparison of the influence of substituents at the C2 position, a significant decrease in cell growth inhibition was observed for naphthoimidazole with a naphthyl ring at C2 (**IM3**), suggesting that the phenyl substituent at the C2 position of the naphthoimidazole ring is relevant for the evaluated activity.

Against glioblastoma cells (SNB-19), the most cytotoxic compound was **IM5** (IC_50_ 21.05 μM). As for HCT-116 cells and leukemia cells (HL-60), the most active naphth[1,2-*d*]imidazoles were **IM6** and **IM4**, with IC_50_ of 21.12 and 8.71 μM, respectively. Comparing the three most cytotoxic imidazoles (**IM4**, **IM5**, and **IM6**) for each cancer cell line tested, it was observed that all of them presented as a substituent at the C2 position a 4-substituted phenyl ring with an electronegative group: -OH, -N(CH_3_)_2,_ and NO_2_, respectively. Thus, it is possible to suggest that the substituted phenyl group located at the C2 carbon of the naphthoimidazole ring improves cytotoxicity activity and promotes selectivity.

In addition, for the cytotoxic activity of **IM6** and **IM7** (Table 2) for each cancer cell line tested, it can be seen that the 2-nitrophenyl substituent group at the C2 carbon of the naphthoimidazole ring makes the compound less cytotoxic to the evaluated tumor cell lines. Comparing the cytotoxic activity against three cancer cell lines, the tested naphthoimidazoles showed to be more active against the leukemia cell line (HL-60), presenting IC_50_ between 8.71 and 29.92 μM. Among them, **IM4** was the most active compound, with an emphasis on its high selectivity for leukemic cells (SI > 41.67).

By considering that high ε_Abs_ values combined with large Δ_ST_ are desirable for fluorescent probes [43] and the photophysical and cytotoxic properties of each naphth[1,2-*d*]imidazole, one can infer that **IM3** (fluorescence intensity 1.41 × 10^6^ au, ε_Abs_ 1.57 × 10^4^ M^−1^ cm^−1^ and Δ_ST_ 103 nm) fits as a good fluorescent probe; however, it showed low cytotoxicity on the cell lines tested. One can also infer that **IM2** (fluorescence intensity 2.49 × 10^6^ au; ε_Abs_ 2.43 × 10^4^ M^−1^ cm^−1^ and Δ_ST_ 60 nm) has photophysical properties that qualify it as a fluorescent probe, and it has cytotoxicity against HCT-116 (IC_50_ 21.69 µM) and selectivity (SI 4.83).

**IM4** stands out for cytotoxicity against HL-60 (IC_50_ 8.71 µM) and selectivity (SI > 41.70); at the same time, it appears to have a high intensity of fluorescence (1.36 × 10^6^ au) and moderate ε_Abs_ (1.48 × 10^4^ M^−1^ cm^−1^) and Δ_ST_ 49 nm. **IM5** was cytotoxic to SNB-19 (IC_50_ 21.05 µM and SI 2.51) and showed promising photophysical properties (fluorescence 7.04 × 10^5^ au; ε_Abs_ 2.71 × 10^4^ M^−1^ cm^−1^ and Δ_ST_ 54 nm).

## 3. Materials and Methods

### 3.1. Materials

All chemicals were purchased from commercial suppliers and used without further purification. Melting points were determined through a PFM-II (Instrumentation MS Tecnopon^®^) melting-point apparatus. The purity of the compounds synthesized was determined by thin-layer chromatography (TLC) using several solvent systems of different polarities. Purification of these compounds was done by column chromatography. Infrared (IR) spectra were recorded on a PerkinElmer (model 10.4.00) spectrophotometer equipped with an Attenuated Total Reflectance ATR sampling unit. NMR spectra were recorded on a Bruker Ascend 400 spectrometer, operating at 400 MHz for ^1^H NMR and 100 MHz for ^13^C NMR. CDCl_3_ and DMSO-d*_6_* were used as solvents with tetramethylsilane (TMS) as the internal standard; chemical shifts (δ) are given in ppm and coupling constants (J) in Hz. Mass spectra were recorded with a Bruker Daltonics (TOF-Q-II) spectrometer using electrospray ionization. UV–vis absorption spectra were obtained using a Hach/Lange spectrophotometer (model DR 5000). Fluorescence emission spectra were obtained using the ISS spectrofluorometer (model PC1).

### 3.2. Synthesis of Naphth[1,2-d]imidazoles ***IM1***–***IM7***

#### 3.2.1. Synthesis of β-Lapachone 2

Lapachol **1** was extracted from the wood of a plant of the genus *Tabebuia* and used after purification and identification, as described previously [44]. The access was registered in the National System of Genetic Heritage and Associated Traditional Knowledge (SISGEN) under the A5FDA89. Yield: 1.5% (m/m). Yellow solid, mp: 138.3–140.3 °C (Lit 139.0–141.0 °C) [45].

In a 25 mL reaction flask, the lapachol (484 mg, 2 mmol) was weighed and incorporated into a concentrated sulfuric acid (H_2_SO_4_) solution (5 mL). The reaction mixture was stirred at room temperature for 1.0 h, then poured into 400 mL of ice-cold deionized water. The solid obtained was vacuum filtered and allowed to dry at room temperature [44], which resulted in a yield of 95%. Orange solid, mp: 155 °C (Lit 154–155 °C) [46]. ^1^H NMR (400 MHz, DMSO-d*_6_*) δ [ppm]: 7.91 (d, *J* = 7.6 Hz, 1H), 7.77 (m, 2H), 7.61 (m, 1H), 2.40 (t, *J* = 6.6 Hz, 2H), 1.82 (t, *J* = 6.6 Hz, 2H), 1.43 (s, 6H). ^13^C NMR (100 MHz DMSO-d*_6_*) δ (ppm): 179.1, 177.8, 160.6, 135.0, 132.1, 130.8, 129.9, 127.8, 123.7, 112.5, 79.0, 30.8, 26.3, 15.9.

#### 3.2.2. General Synthesis of the Naphth[1,2-*d*]imidazoles

The solution of β-Lap **2** (242 mg; 1.0 mmol) was prepared in glacial acetic acid (6 mL), and was added aldehyde adequate (2.5 mmol). The reaction mixture was placed at a temperature of 70 °C and added to ammonium acetate (1.27 g; 16.5 mmol) that was divided into three parts and remained at this temperature under stirring until the end of the reaction [10]. The reactions were followed by Thin Layer Chromatography (TLC), and the reaction times varied in the range of 30 min to 4 h. In experiments using 4-dimethylaminobenzaldehyde and 4-nitrobenzaldehyde, there was a precipitate formation in the reaction. However, in experiments employing formaldehyde, benzaldehyde, 1-naphthaldehyde, 4-hydroxybenzaldehyde, and 2-nitrobenzaldehyde, there was no precipitate formation in the reaction. Then, after the reaction time, the reaction mixture was poured into a cold solution of 5.0% (*m/v*) of NaHSO_3_ for precipitate formation. The solid was filtered and washed with a solution of 5.0% (*m/v*) of NaHCO_3_, and water was deionized at neutral pH and dried at room temperature.

##### Synthesis of 4,5-dihydro-6,6-dimethyl-6*H*-2-pyran[*b*-4,3]naphth[1,2-*d*]imidazole (**IM1**)

The reaction was heated to 70 °C for 4.0 h. Compound **IM1** was obtained as a yellow crystalline solid (131 mg, 0.519 mmol, yield: 52.0%), mp: 255–259 °C. ^1^H NMR (400 MHz, DMSO-d*_6_*) δ [ppm]: 8.30 (d, *J* = 8.0 Hz, 1H), 8.17 (s, 1H), 8.12 (d, 1H), 7.54 (t, 1H), 7.42 (t, 1H), 2.98 (t, 2H), 1.94 (t, 2H), 1.40 (s, 6H). ^13^C NMR (100 MHz, DMSO-d*_6_*) δ [ppm]: 143.9, 132.9, 138.4, 127.7, 125.7, 124.1, 123.3, 122.7, 122.1, 121.0, 104.4, 74.2, 31.5, 26.5, 18.5. IR (KBr) ʋ_max_/cm^−1^: 3435, 3088, 2971, 2925, 2841, 1608, 1538, 1484, 1453, 1362, 1252, 1164, 1122, 1054, 947, and 770, Figure S1.

##### Synthesis of 4,5-dihydro-6,6-dimethyl-6*H*-2-(phenyl)-pyran[*b*-4,3]naphth[1,2-*d*]imidazole (**IM2**)

The reaction was heated to 70 °C for 1.0 h. Compound **IM2** was obtained as a light-yellow solid (60 mg, 0.183 mmol, yield: 18.3%), mp: 278–279 °C. ^1^H NMR (400 MHz, DMSO-d*_6_*) δ [ppm]: 13.24 (s, 0.3H), 12.76 (s, 0.5H), 8.37–8.46 (m, 1H), 8.20–8.29 (m, 2H), 8.10–8.20 (m, 1H), 7.51–7.63 (m, 3H), 7.39-7.51 (m, 2H), 3.00–3.17(m, 2H), 1.87–2.06 (m, 2H), 1.43 (s, 6H). ^13^C NMR (100 MHz, DMSO-d*_6_*) δ [ppm]: 147.9, 144.5, 132.2, 131.2, 130.6, 129.1, 128.8, 126.0, 125.9, 125.7, 123.4, 122.8, 122.1, 121.2, 102.4, 74.4, 31.4, 26.5, 18.8. IR (KBr) ʋ_max_/cm^−1^: 3432, 3067, 2972, 2852, 2928, 1600, 1520, 1256, 1157, and 1056. HRMS (ESI-TOF) calculated for C_22_H_20_N_2_O [M+H]^+^: 329.1609. Found: 329.1646, Figure S2.

##### Synthesis of 4,5-dihydro-6,6-dimethyl-6*H*-2-(naphthalenyl)-pyran[*b*-4,3]naphth[1,2-*d*]imidazole (**IM3**)

The reaction was heated to 70 °C for 2.0 h. Compound **IM3** was obtained as a pale-yellow solid (83 mg, 0.22 mmol, yield: 25.4%). ^1^H NMR (400 MHz, DMSO-d*_6_*) δ [ppm]: 13.39 (s, 0.4H); 12,92 (s, 0.6H); 9.15 (dd, 1H); 8.45 (dd, 1H); 8.19 (d, *J* = 7.9 Hz, 1H); 8.02–8.12 (m, 3H); 7.55–7.74 (m, 4H); 7.41–7.49 (m, 1H); 3.06 (m, 1.2H); 3.18 (m, 0.8H); 2.00 (m, 2H); 1.45 (s, 6H). ^13^C NMR (100 MHz, DMSO-d*_6_*) δ [ppm]: 147.7, 144,5, 133.6, 132.2, 130.6, 130.5, 129.4, 129.2, 128.0, 128.2, 127.5, 126.5, 126.1, 125.8, 125.7, 125.2, 123.3, 122.8, 122.1, 121.1, 102.3, 74.3, 31.4, 26.4, 18.7. IR (KBr) ʋ_max_/cm^−1^: 3405, 3061, 2977, 2929, 1588, 1518, 1257, 1121, and 1054. HRMS (ESI-TOF) calculated for C_26_H_22_N_2_O_2_ [M+H]^+^: 378.1732. Found: 379.1802, Figure S3.

##### Synthesis of 4,5-dihydro-6,6-dimethyl-6*H*-2-(4-hidroxyphenyl)-pyran[*b*-4,3]naphth[1,2-*d*]imidazole (**IM4**)

The reaction was heated to 70 °C for 1.0 h. Compound **IM4** was obtained as a gray amorphous solid (34 mg, 0.099 mmol, yield: 9.9%). ^1^H NMR (400 MHz, DMSO-d*_6_*) δ [ppm]: 12.97 (s, 0.3H), 12.49 (s, 0.5H), 9.85 (s, 1H), 8.32-8.41 (m, 1H), 8.13 (d, 1H), 8.08 (d, 2H), 7.49–7.59 (m, 1H), 7.33–7.44 (m, 1H), 6.92 (d, *J* = 8.2 Hz, 2H), 2.96–3.14 (m, 2H), 1.89–1.98 (m, 2H), 1.42 (s, 6H). ^13^C NMR (100 MHz, DMSO-d*_6_*) δ [ppm]: 158.3, 148.4, 143.9, 131.9, 130.7, 127.5, 125.5, 125.4, 123.0, 121.9, 121.9, 121.7, 121.0, 115.4, 102.3, 74.1, 31.3, 26.4, 18.7. IR (KBr) ʋ_max_/cm^−1^: 3422, 3071, 2974, 2849, 2929,1613, 1533, 1265, 1160, and 1055. HRMS (ESI-TOF) calculated for C_22_H_20_N_2_O_2_ [M+H]^+^: 345.1558. Found: 345.1590, Figure S4.

##### Synthesis of 4,5-dihydro-6,6-dimethyl-6*H*-2-(4-dimethylaminophenyl)-pyran[*b*-4,3]naphth[1,2-*d*]imidazole (**IM5**)

The reaction was heated to 70 °C for 3.0 h. Compound **IM5** was obtained as a light-yellow solid (121 mg, 0.326 mmol, yield: 32.6%), mp: 182-184 °C. ^1^H NMR (400 MHz, DMSO-d*_6_*) δ [ppm]: 12.87 (s, 0.4H), 12.42 (s, 0.5H), 8.30–8.42 (m, 1H), 8.03–8.17 (m, 3H), 7.53 (t, *J* = 7.3 Hz, 1H), 7.38 (t, *J* = 7.6 Hz, 1H), 6.86 (d, *J* = 8.7 Hz, 2H), 2.95–3.14 (m, 8H), 1.93–2,01 (m, 2H), 1.41 (s, 6H). ^13^C NMR (100 MHz, DMSO-d*_6_*) δ [ppm]: 150.7, 150.6, 143.8, 130.8, 127.1, 125.5, 123.0, 122.4, 122.1, 121.2, 120.3, 112.0, 102.5, 74.1, 40.0, 31.5, 26.5, 18.9. IR (KBr) ʋmax/cm^−1^: 3432, 3067, 2979, 2841, 2930, 1610, 1518, 1256, 1159, and 1055. HRMS (ESI-TOF) calculated for C_24_H_25_N_3_O [M+H]^+^: 372.2031. Found: 372.2073, Figure S5.

##### Synthesis of 4,5-dihydro-6,6-dimethyl-6*H*-2-(4-nitrophenyl)-pyran[*b*-4,3]naphth[1,2-*d*]imidazole (**IM6**)

The reaction was heated to 70 °C for 30 min. Compound **IM6** was obtained as a red crystalline solid (183 mg, 0.490 mmol, yield: 49.1%), mp: 259–260 °C. ^1^H NMR (400 MHz, DMSO-d*_6_*) δ [ppm]: 13.61 (s, 0.3H), 13.10 (s, 0.6H), 8.36–8.52 (m, 5H), 8.13–8.22 (m, 1H), 7.56–7.67 (m, 1H), 2.96–3.16 (m, 2H), 1.90–2.09 (m, 2H), 1.42, 144 (s, 6H). ^13^C NMR (100 MHz, DMSO-d*_6_*) δ [ppm]: 146.8, 146.6, 144.2, 136.6, 133.0, 132.1, 126.6, 126.3, 125.7, 124.3, 124.0, 123.2, 122.3, 121.2, 102.2, 74.7, 31.3, 26.5, 18.7. IR (KBr) ʋ_max_/cm^−1^: 3348, 2975, 2845, 2929, 1604, 1511, 1258, 1155, and 1057. HRMS (ESI-TOF) calculated for C_22_H_19_N_3_O_3_ [M+H]^+^: 374.1460. Found: 374.1511, Figure S6.

##### Synthesis of 4,5-dihydro-6,6-dimethyl-6*H*-2-(2-nitrophenyl)-pyran[*b*-4,3]naphth[1,2-*d*]imidazole (**IM7**)

The reaction was heated to 70 °C for 2.5 h. Compound **IM7** was obtained as a red solid (194 mg, 0.520 mmol, yield: 52.0%), mp: 139-141 °C. ^1^H NMR (400 MHz, DMSO-d*_6_*) δ [ppm]: 13.52 (s, 0.3H), 13.08 (s, 0.7H), 8.27 (d, 1H), 8.20–8.11 (m, 1H), 8.03 (t, *J* = 8.7 Hz, 2H), 7.83–7.92 (m, 1H), 7.68–7.77 (m, 1H), 7.53–7.63 (m, 1H), 7.41–7.50 (m, 1H), 2.99 (t, *J* = 6.5 Hz, 2H), 1.99 (t, *J* = 6.6 Hz, 2H), 1.43, 1.41 (s, 6H). ^13^C NMR (100 MHz, DMSO-d*_6_*) δ [ppm]: 148.8, 144.7, 143.8, 132.4, 132.6, 131.2, 130.9, 130.3, 126.1, 125.6, 124.6, 124.4, 123.7, 123.2, 122.2, 121.1, 102.2, 74.6, 31.4, 26.5, 18.7. IR (KBr) ʋ_max_/cm^−1^: 3415, 3116, 2973, 2850, 2923, 1602, 1521, 1260, 1162, and 1057. HRMS (ESI-TOF) calculated for C_22_H_19_N_3_O_3_ [M+H]^+^: 374.1460. Found: 374.1495, Figure S7.

### 3.3. Evaluation of Photophysical Properties

#### 3.3.1. Obtaining Visible Ultraviolet Absorption Spectra

A stock solution in dichloromethane (CH_2_Cl_2_) of each compound was prepared at a concentration of 4000 µM. From the stock solution, solutions were prepared at a concentration of 20 µM of each compound in four different solvents: hexane, CH_2_Cl_2_, dimethyl sulfoxide (DMSO), and methanol (CH_3_OH). Then, measurement in the range of 190 to 800 nm was performed, with the wavelengths of maximum absorption (λ_max_) of the compounds in the different solvents shown in Table S1 and Figure S8.

#### 3.3.2. Molar Absorptivity Coefficient

The molar absorptivity coefficient (ε_Abs_) was determined using an equation applied by Lambert–Beer (ε_Abs_ = A/LxC, where A—maximum absorbance; L—the optical path of the cuvette used (1 cm); and C—concentration of the analyzed sample in M).

#### 3.3.3. Fluorescence Emission Spectrum and Stokes Shift

Stock solutions used for each compound, at a concentration of 4000 µM, in the solvents in which the sample showed better resolution of the maximum absorption band, were DMSO for **IM2**, CH_3_OH for **IM6**, hexane for **IM7**, and CH_2_Cl_2_ for the others. The stock solutions were diluted to a concentration of 20 µM, and readings used the excitation wavelength of 345 nm for all compounds. The Stokes shift (Δ_ST_) was calculated from the difference between the absorbance and excitation wavelengths (λ_Abs_–λ_Emis_), Figure S8.

### 3.4. Evaluation of the Cytotoxic Activity

#### 3.4.1. Cell Lines

Brain tumor (SNB-19), human colorectal carcinoma (HCT-116), and human promyelocytic leukemia (HL-60) cells were obtained from the National Cancer Institute (NCI) (Bethesda, MD, USA). The L929 cells (mouse fibroblast L cells NCTC clone 929) were purchased from the American Type Culture Collection (Manassas, VA, USA). The cells were grown on RPMI 1640 medium supplemented with 10% fetal bovine serum and 1% antibiotics (penicillin/streptomycin) at 37 °C with 5% CO_2_.

#### 3.4.2. Assessment of In Vitro Anticancer Activity

Cytotoxic potential of the naphth[1,2-*d*]imidazoles **IM1**–**IM7** was assessed after 72 h of exposure to the tumor cell lines of human SNB-19, HCT-116, HL-60, and normal cell line L929. Cells were plated in 96-well plates (0.7 × 10^5^ cells/well for SNB-19, 0.3 × 10^6^ cells/well for HCT-116, and 0.3 × 10^6^ cells/well for HL-60). Compounds were dissolved with DMSO at concentrations in the 0.078–10 μg.mL^−1^ range. Doxorubicin (0.001–1.10 μM) was used as the positive control, and negative control groups received the same amount of vehicle (DMSO). The cell viability was determined by the reduction of the yellow dye 3-(4,5-dimethyl-2-thiazol)-2,5-diphenyl-2H-tetrazolium bromide (MTT) to a blue formazan product [35]. At the end of the incubation time (69 h), the plates were centrifuged, and the medium was replaced with fresh medium (200 μL) containing 0.5 mg/mL of MTT. Three hours later, the MTT formazan product was dissolved in DMSO (150 μL), and the absorbance was measured using a multi-plate reader (Spectra Count, Packard, ON, Canada). The drug effect was quantified as the percentage of control absorbance of the reduced dye at 550 nm. All experiments were performed in three independent assays, and the half maximal inhibitory concentration (IC_50_) and their 95% confidence intervals were achieved by nonlinear regression.

## 4. Conclusions

Naphth[1,2-*d*]imidazoles **IM1**–**IM7** showed high levels of cytotoxic activity and selectivity against the tested cancer cells and promising optical properties. The cytotoxicity results and photophysical properties presented by naphth[1,2-*d*]imidazoles **IM2**, **IM3**, **IM4**, and **IM5** qualify them for further studies in the development of fluorescent anticancer probes using this scaffold, making possible the use of naphth[1,2-*d*]imidazoles as fluorescent probes/therapeutic molecules in theranostic systems for cancer treatment/diagnosis.

## Figures and Tables

**Table 2:** Cytotoxic activity of **IM1**, **IM2**, **IM4**, **IM5**, **IM6**, and **IM7** after a 72 h exposure expressed by IC_50_ (µM) and confidence interval.

	Cell Line						
Compound	L929	SNB-19		HCT-116		HL-60	
	IC_50_ (μM) ^a^	IC_50_ (μM) ^a^	SI ^b^	IC_50_ (μM) ^a^	SI ^b^	IC_50_ (μM) ^a^	SI ^b^
**IM1**	186.17	46.44 (38.59–55.76)	4.00	62.11 (54.14–71.31)	3.00	24.23 (22.32–21.41)	7.68
**IM2**	104.85	26.97 (24.44–26.96)	3.89	21.69 (18.34–25.66)	4.83	29.92 (14.44–19.87)	3.50
**IM4**	>363.21	31.90 (28.39–35.83)	>11.38	23.04 (21.56–24.67)	>15.76	8.71 (7.46–10.22)	>41.70
**IM5**	52.84	21.05 (18.20–24.39)	2.51	44.07 (37.42–51.87)	1.20	12.35 (10.22–14.55)	4.28
**IM6**	151.55	22.27 (20.23–24.49)	6.81	21.12 (19.56–22.78)	7.18	13.67 (14.79–18.09)	11.09
**IM7**	NE	>67.00	NE	35.11 (28.94–42.56)	NE	14.90 (10.64–14.17)	NE
Doxorubicin	3.16	2.21 (1.90–2.56)	1.43	0.20 (0.15–0.26)	15.80	0.04 (0.035–0.039)	79.00

NE—not evaluated. ^a^: IC_50_ is the concentration at which 50% of cells were undergoing cytotoxic cell death due to synthesized compound treatment. ^b^: SI (selectivity index) equals the ratio of IC_50_ for fibroblasts L929/IC_50_ for the cancer cell lines.

## Data Availability

Data is contained within the article or Supplementary Material.

## Supplementary Materials

The following supporting information can be downloaded at https://www.mdpi.com/article/10.3390/molecules28073008/s1. Figure S1: NMR spectra of 4,5-dihydro-6,6-dimethyl-6*H*-2-pyran[*b*-4,3]naphth[1,2-*d*]imidazole (**IM1**) in DMSO-d*_6_*; Figure S2: NMR spectra of 4,5-dihydro-6,6-dimethyl-6*H*-2-(phenyl)-pyran[*b*-4,3]naphth[1,2-*d*]imidazole (**IM2**) in DMSO-d*_6_* and ESI-MS; Figure S3: NMR spectra of 4,5-dihydro-6,6-dimethyl-6*H*-2-(naphthalenyl)-pyran[*b*-4,3]naphth[1,2-*d*]imidazole (**IM3**) in DMSO-d*_6_*and ESI-MS; Figure S4: NMR spectra of 4,5-dihydro-6,6-dimethyl-6*H-*2-(4-hidroxyphenyl)-pyran[*b*-4,3]naphth[1,2-*d*]imidazole (**IM4**) in DMSO-d*_6_* and ESI-MS; Figure S5: NMR spectra of 4,5-dihydro-6,6-dimethyl-6*H*-2-(4-dimethylaminophenyl)-pyran[*b*-4,3]naphth[1,2-*d*]imidazole (**IM5**) in DMSO-d*_6_* and ESI-MS; Figure S6: NMR spectra of 4,5-dihydro-6,6-dimethyl-6H-2-(4-nitrophenyl)-pyran[*b*-4,3]naphth[1,2-*d*]imidazole (**IM6**) in DMSO-d_6_ and ESI-MS; Figure S7: NMR spectra of 4,5-dihydro-6,6-dimethyl-6*H*-2-(2-nitrophenyl)-pyran[*b*-4,3]naphth[1,2-*d*]imidazole (**IM7)** in DMSO-d*_6_*and ESI-MS; Figure S8: Absorbance and emission spectra of naphthoimidazoles **IM1**–**IM7**; Table S1: Wavelength (nm) and absorbance of the scanning spectra of the naphth[1,2-*d*]imidazoles obtained in the solvatochromism study.

## Sample Availability

Samples of the compounds are available from the authors.
