# Supplementary material for: Naphth[1,2-d]imidazoles Bioactive from β-Lapachone: Fluorescent Probes and Cytotoxic Agents to Cancer Cells
Source: Molecules. 2023 Mar 28;28(7):3008. doi: 10.3390/molecules28073008 (PMC10096064; doi:10.3390/molecules28073008)
Supplement: Supplementary file 1 [file molecules-28-03008-s001.zip › molecules-2272364-supplementary.pdf]

SUPPLEMENTARY INFORMATION

Naphth[1,2-*d*]imidazoles bioactive from β-Lapachone: fluorescent probes and cytotoxic agents to cancer cells

Victória Laysna dos Anjos Santos [1], Arlan de Assis Gonsalves [1,2], Délis Galvão Guimarães [3], Sidney Silva Simplicio [1], Helinando Pequeno de Oliveira [2], Lara Polyana Silva Ramos [4], Marcília Pinheiro da Costa [4], Fátima de Cássia Evangelista de Oliveira [5], Claudia Pessoa [5], Cleônia Roberta Melo Araújo [1,*]

1. Molecular structure elucidation of synthesized naphth[1,2-*d*]imidazoles (IM1–IM7).

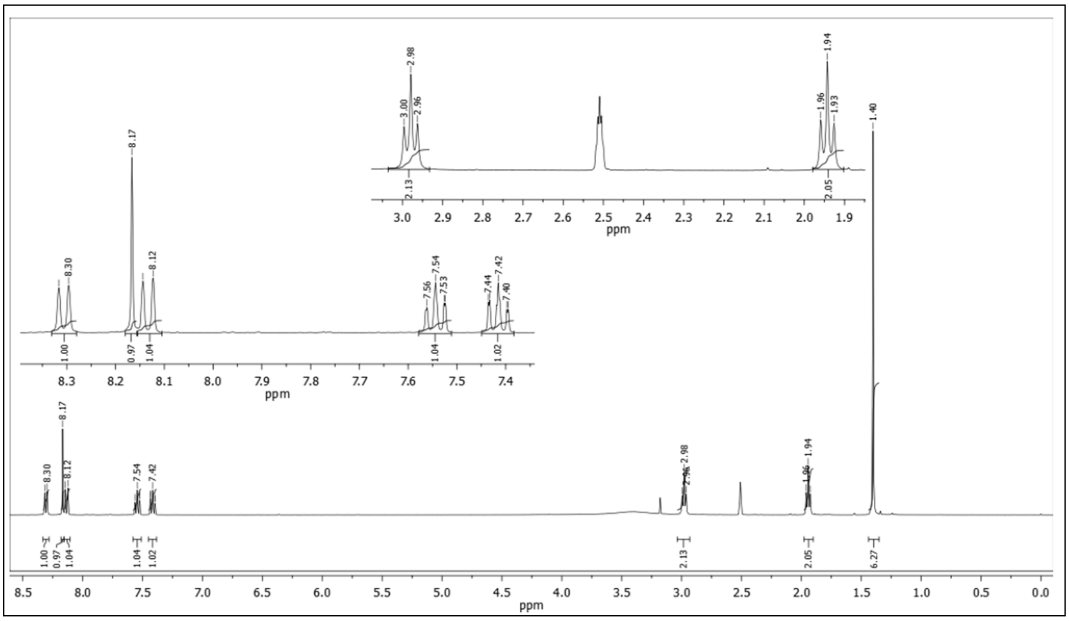

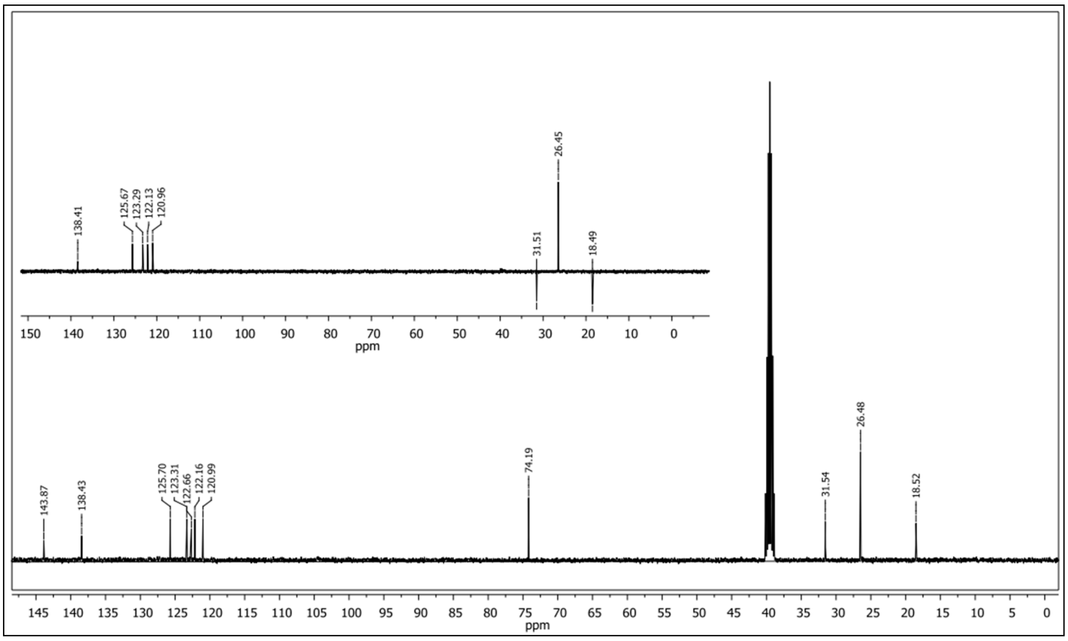

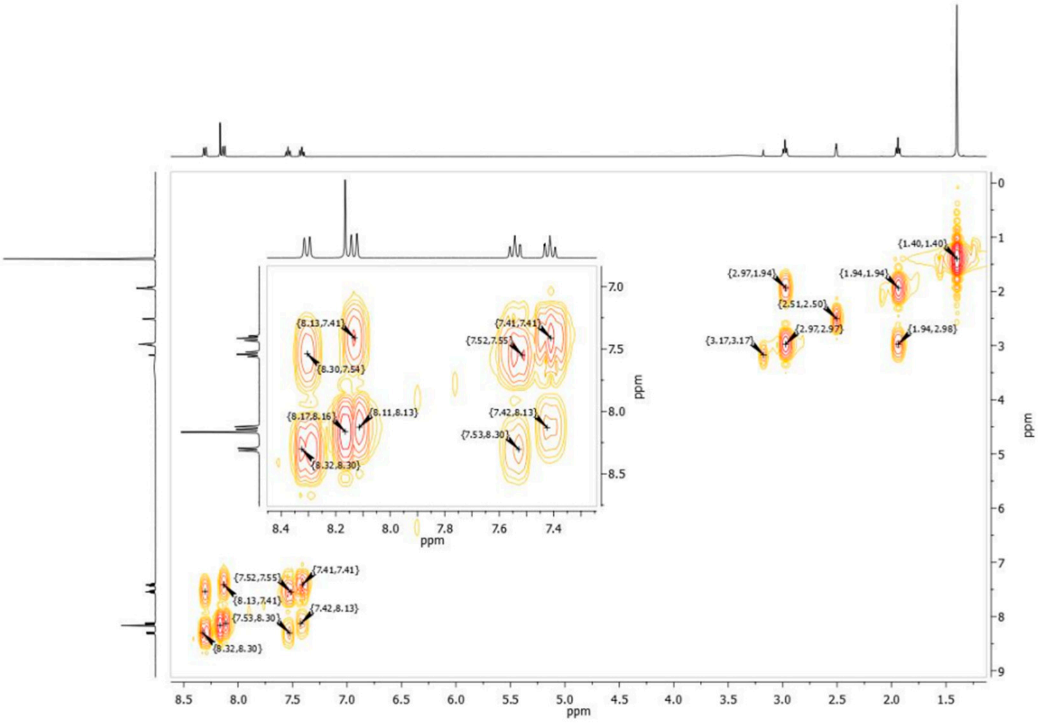
{1.40,1.40}
{2.97,1.94}
{1.94,1.94}
{2.51,2.50}
{3.17,3.17}
{2.97,2.97}
{1.94,2.98}
{8.13,7.41}
{8.30,7.54}
{7.41,7.41}
{7.52,7.55}
{8.17,8.16}
{8.11,8.13}
{8.32,8.30}
{7.42,8.13}
{7.53,8.30}
{7.52,7.55}
{7.41,7.41}
{8.13,7.41}
{7.53,8.30}
{7.42,8.13}
{8.32,8.30}
ppm
ppm
ppm

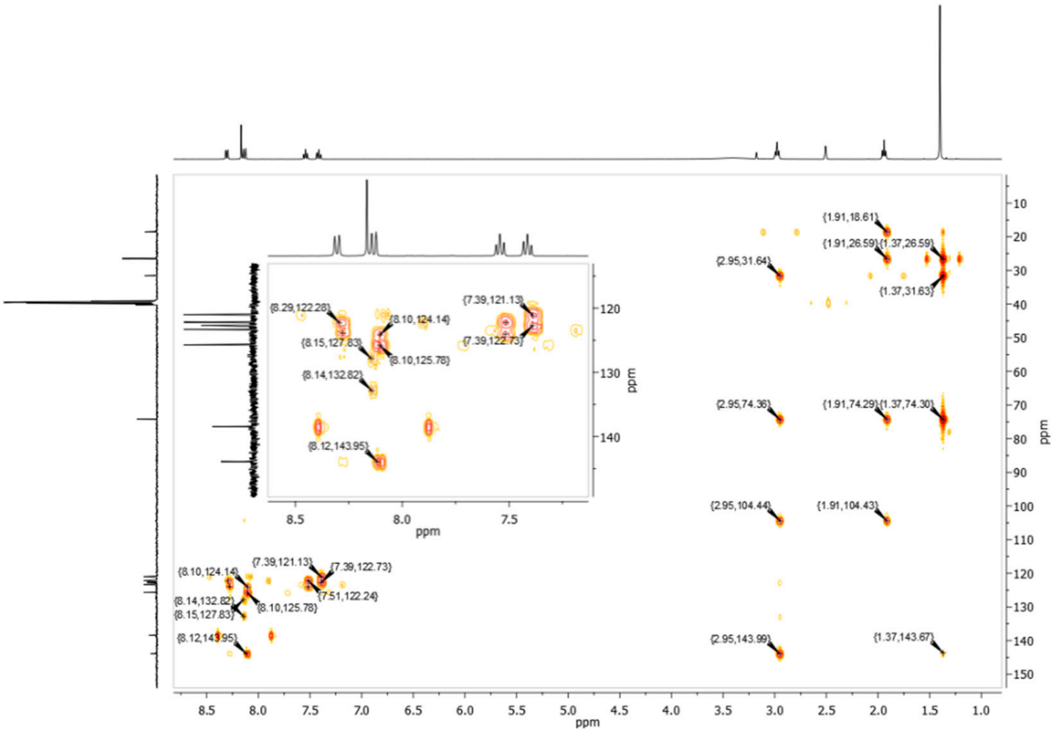
{1.91,18.61}
{1.91,26.59}
{1.37,26.59}
{2.95,31.64}
{1.37,31.63}
{8.29,122.28}
{8.10,124.14}
{7.39,121.13}
{8.15,127.83}
{8.10,125.78}
{7.39,122.73}
{8.14,132.82}
{8.12,143.95}
{2.95,74.36}
{1.91,74.29}
{1.37,74.30}
{2.95,104.44}
{1.91,104.43}
{8.10,124.14}
{7.39,121.13}
{7.39,122.73}
{8.14,132.82}
{8.15,127.83}
{8.10,125.78}
{7.51,122.24}
{8.12,143.95}
{2.95,143.99}
{1.37,143.67}
ppm
ppm
ppm

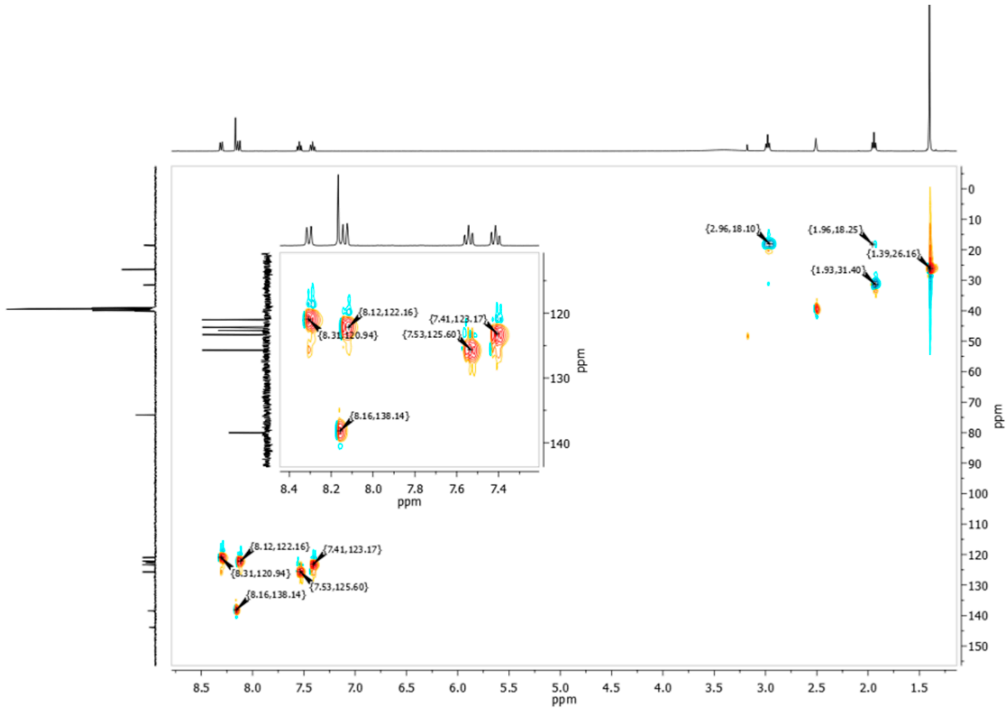

Figure S1. NMR spectra of 4,5-dihydro-6,6-dimethyl-6*H*-2-pyran[*b*-4,3]naphth[1,2-*d*]imidazole (**IM1**) in DMSO-*d*6.

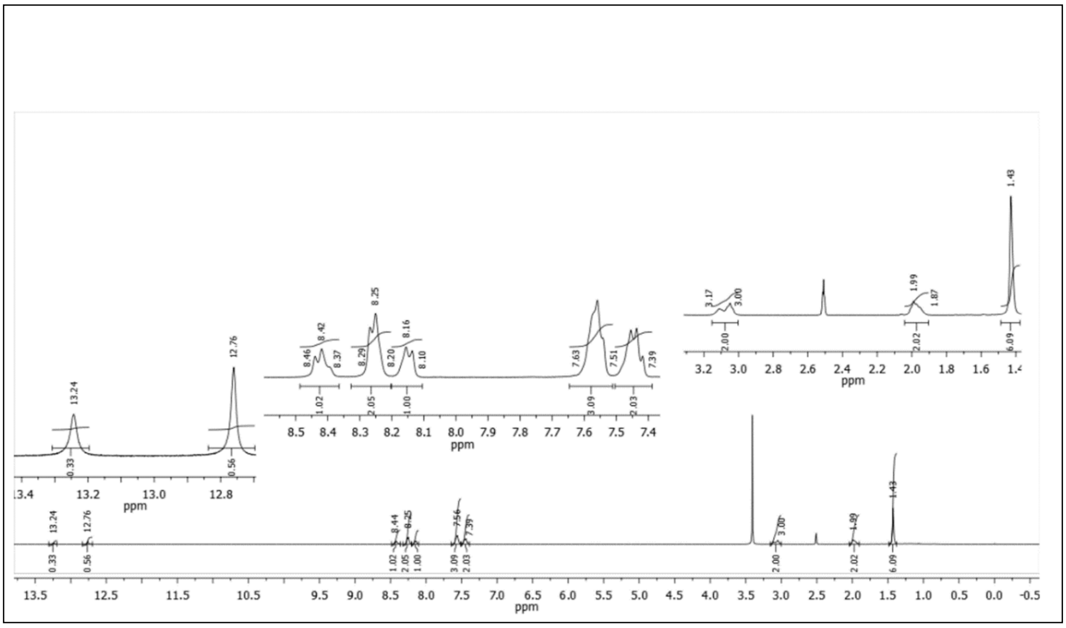
13.24
12.76
8.46
8.42
8.37
8.29
8.25
8.20
8.16
8.10
7.63
7.51
7.39
3.17
3.00
1.99
1.87
1.43
ppm

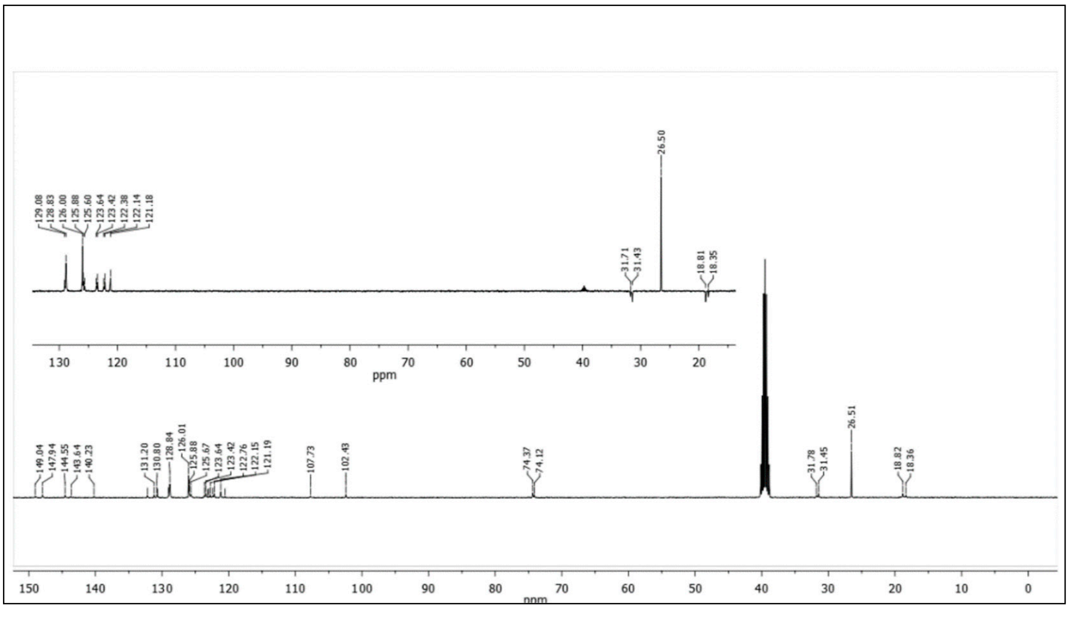
26.50
31.71
31.43
18.81
18.35
149.04
147.94
144.55
143.64
140.23
131.20
130.80
128.84
126.01
125.88
125.67
123.64
123.42
122.76
122.15
121.19
107.73
102.43
74.37
74.12
31.78
31.45
26.51
18.82
18.36
ppm

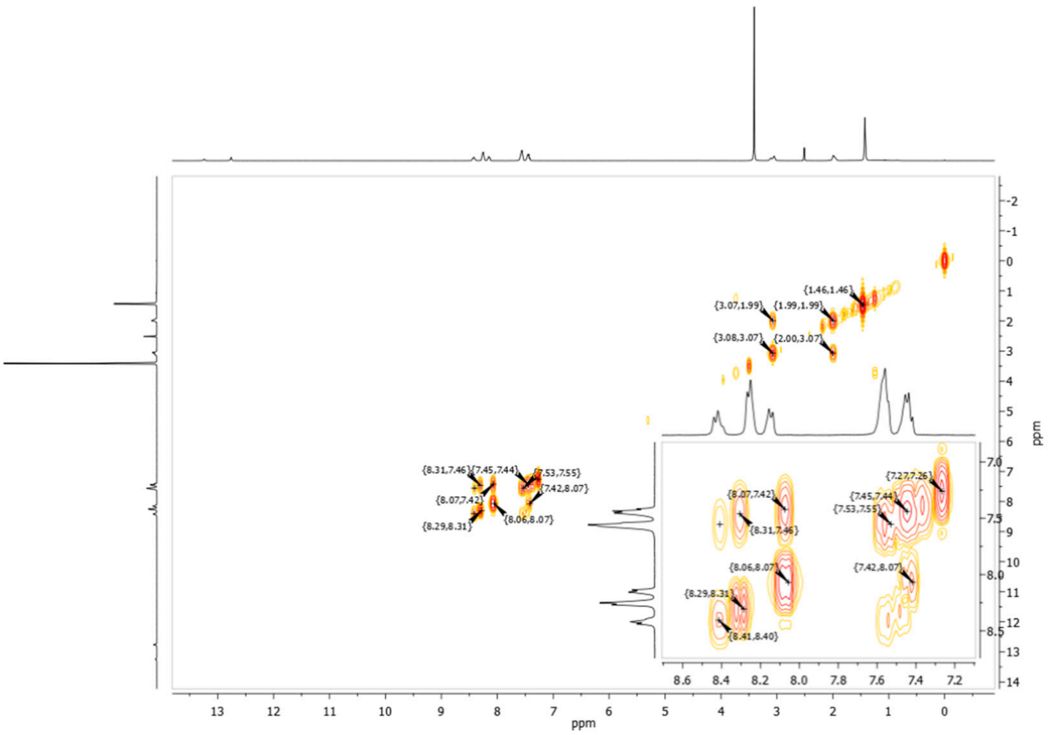
{1.46,1.46}
{3.07,1.99}
{1.99,1.99}
{3.08,3.07}
{2.00,3.07}
{8.31,7.46}
{7.45,7.44}
{7.53,7.55}
{7.42,8.07}
{8.07,7.42}
{8.06,8.07}
{8.29,8.31}
{7.27,7.26}
{8.31,7.46}
{8.41,8.40}
ppm

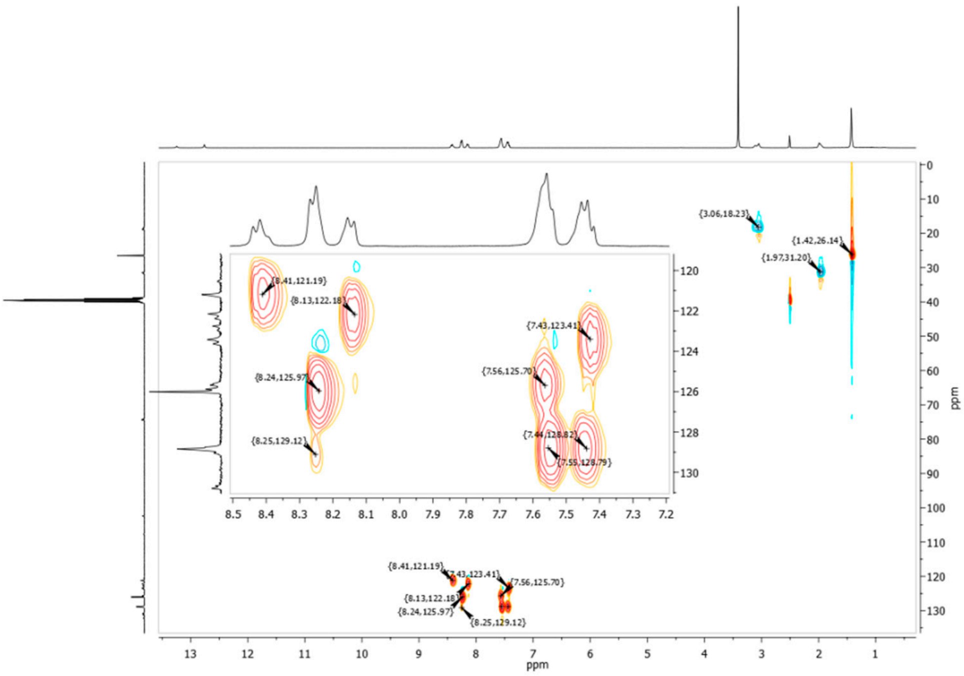
{3.06,18.23}
{1.42,26.14}
{1.97,31.20}
{8.41,121.19}
{8.13,122.18}
{7.43,123.41}
{8.24,125.97}
{7.56,125.70}
{7.44,128.82}
{7.55,128.79}
{8.25,129.12}
ppm

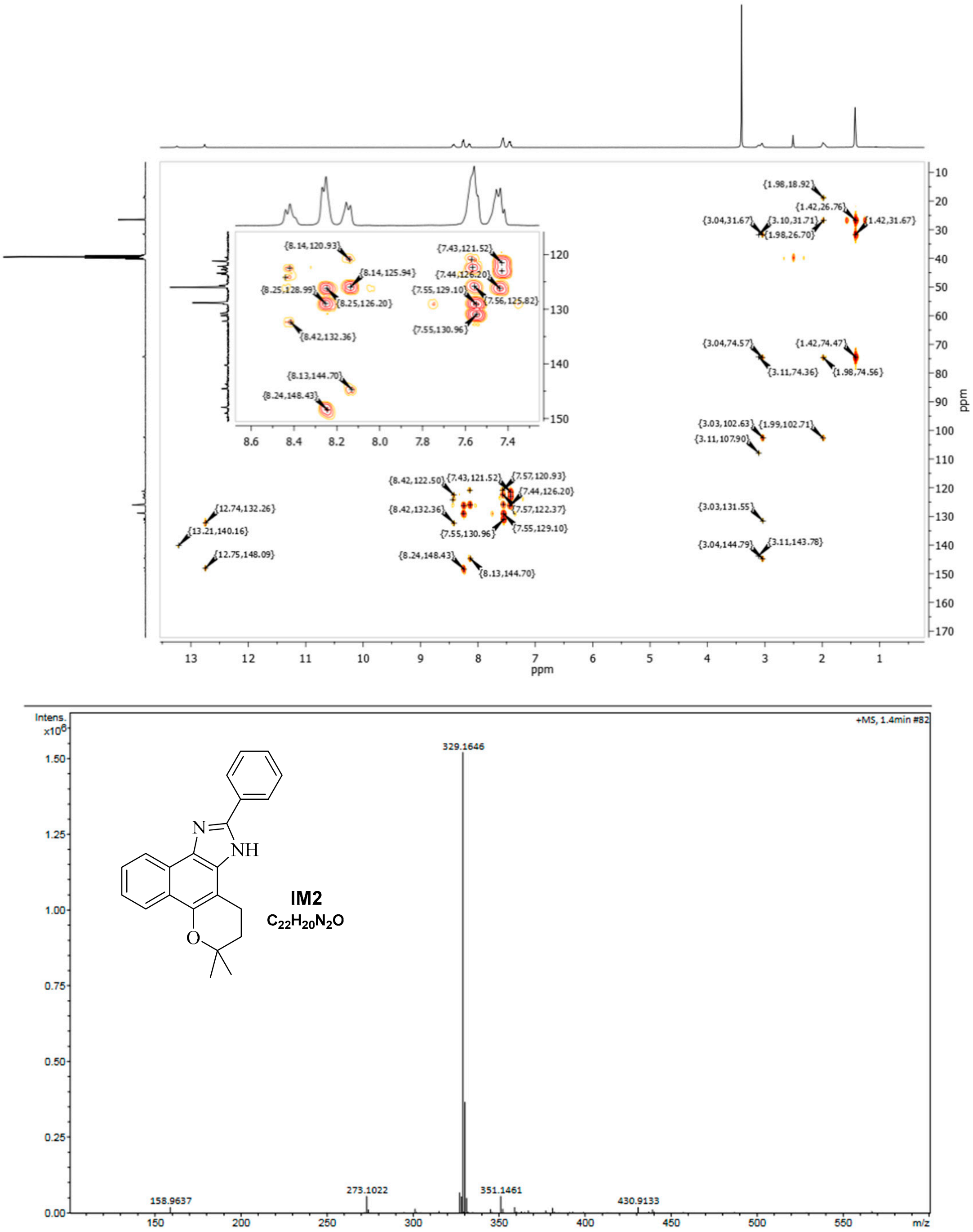

Figure S2. NMR spectra of 4,5-dihydro-6,6-dimethyl-6*H*-2-(phenyl)-pyran[*b*-4,3]naphth[1,2-*d*]imidazole (**IM2)** in DMSO-d_6 and ESI-MS.

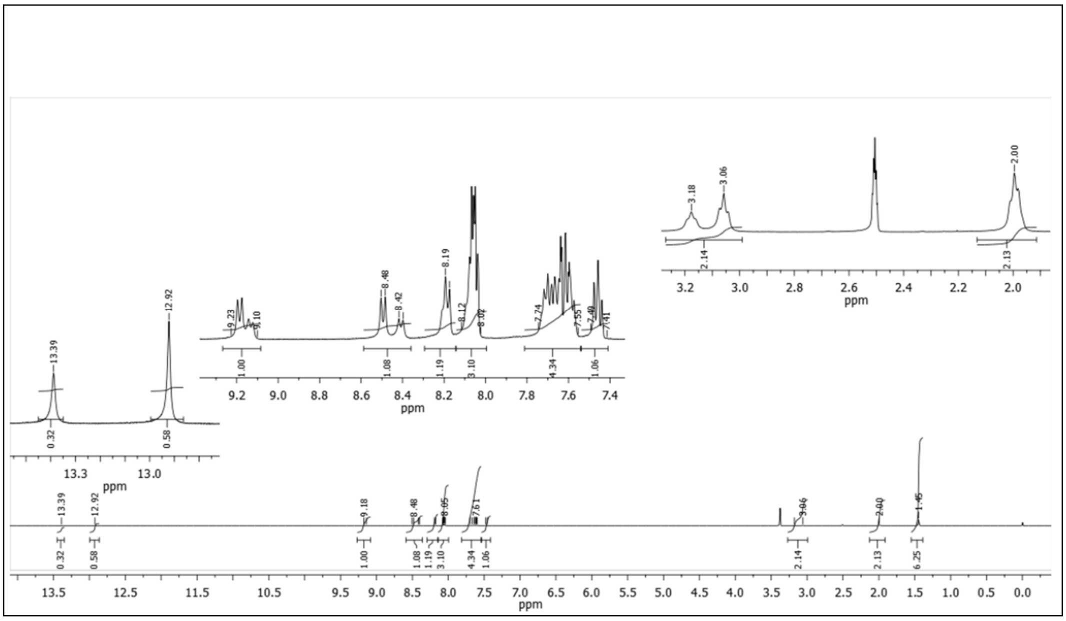

ppm

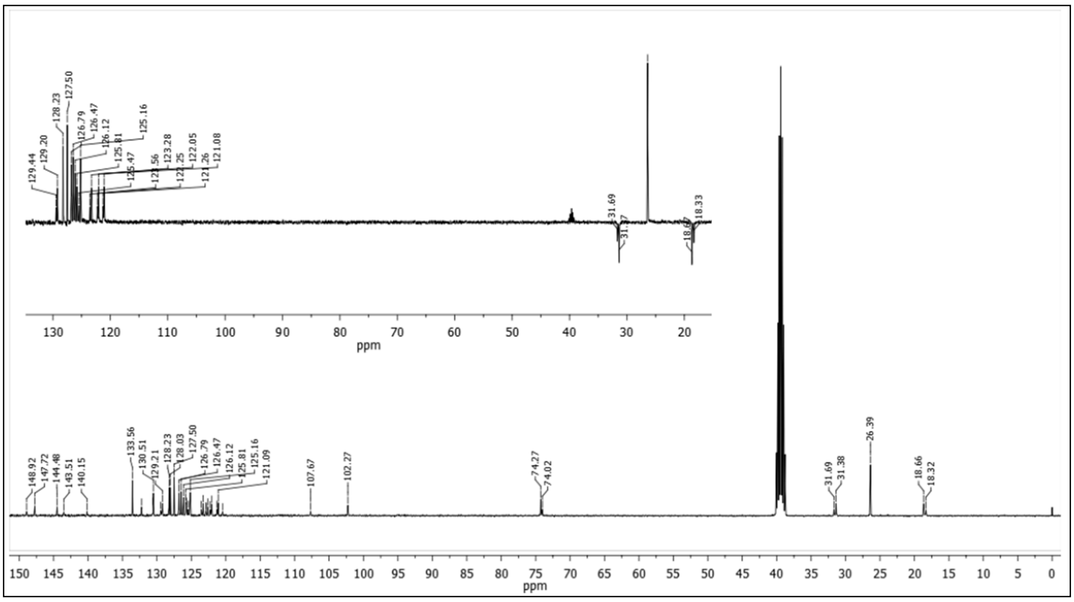

ppm

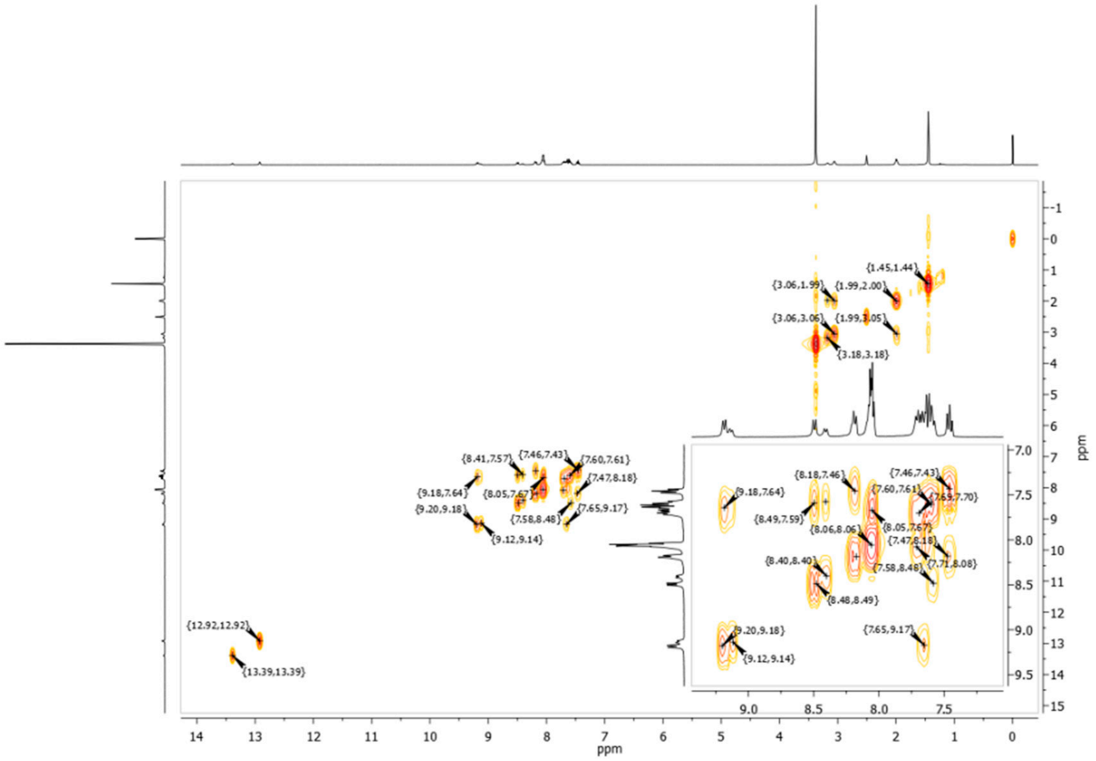
{1.45,1.44}
{3.06,1.99}
{1.99,2.00}
{3.06,3.06}
{1.99,3.05}
{3.18,3.18}
{8.41,7.57}
{7.46,7.43}
{7.60,7.61}
{7.47,8.18}
{9.18,7.64}
{8.05,7.67}
{9.20,9.18}
{7.65,9.17}
{7.58,8.48}
{9.12,9.14}
{12.92,12.92}
{13.39,13.39}
{8.18,7.46}
{7.65,7.70}
{8.49,7.59}
{8.06,8.06}
{8.40,8.40}
{7.71,8.08}
{8.48,8.49}
ppm

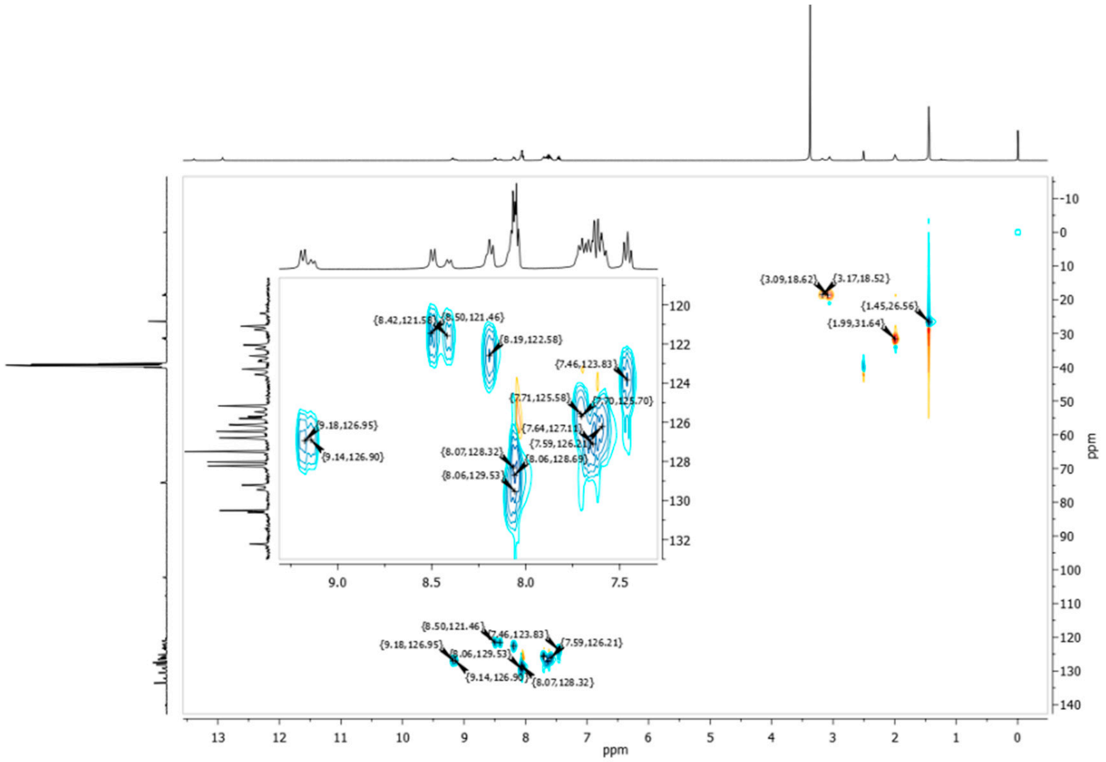
{8.42,121.58}
{8.50,121.46}
{8.19,122.58}
{7.46,123.83}
{7.71,125.58}
{7.70,125.70}
{9.18,126.95}
{7.64,127.11}
{7.59,126.21}
{9.14,126.90}
{8.07,128.32}
{8.06,128.69}
{8.06,129.53}
{3.09,18.62}
{3.17,18.52}
{1.45,26.56}
{1.99,31.64}
{8.50,121.46}
{7.46,123.83}
{7.59,126.21}
{9.18,126.95}
{8.06,129.53}
{9.14,126.90}
{8.07,128.32}
ppm

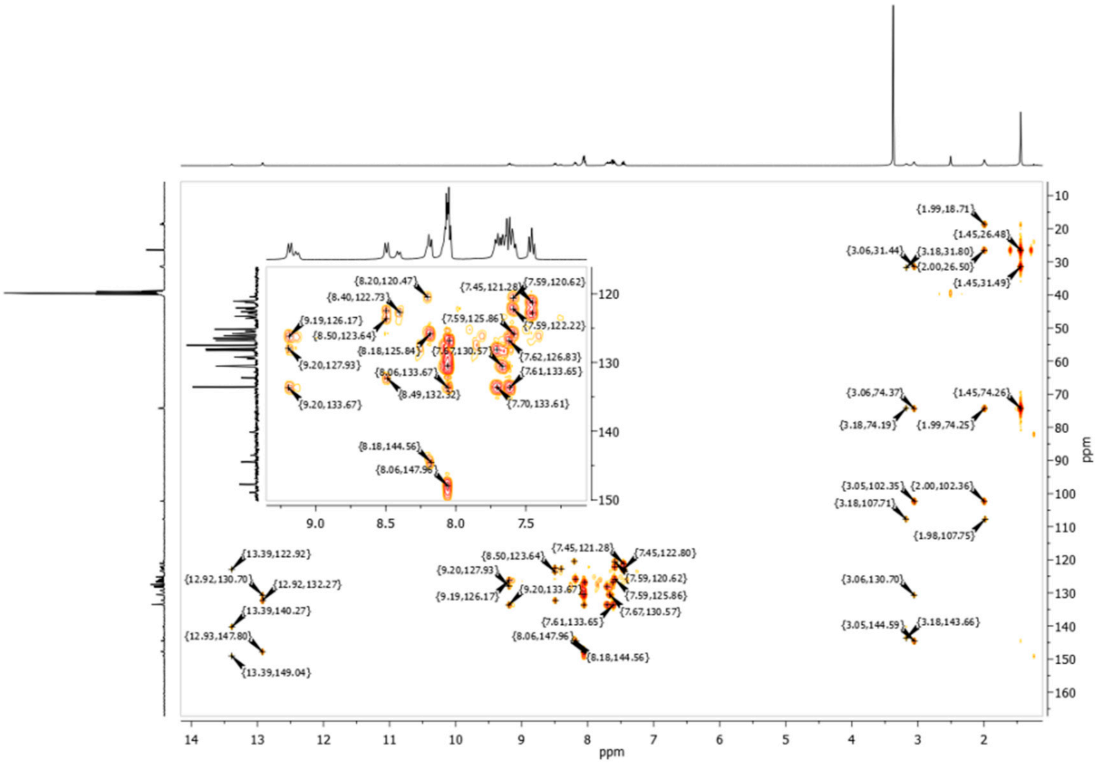

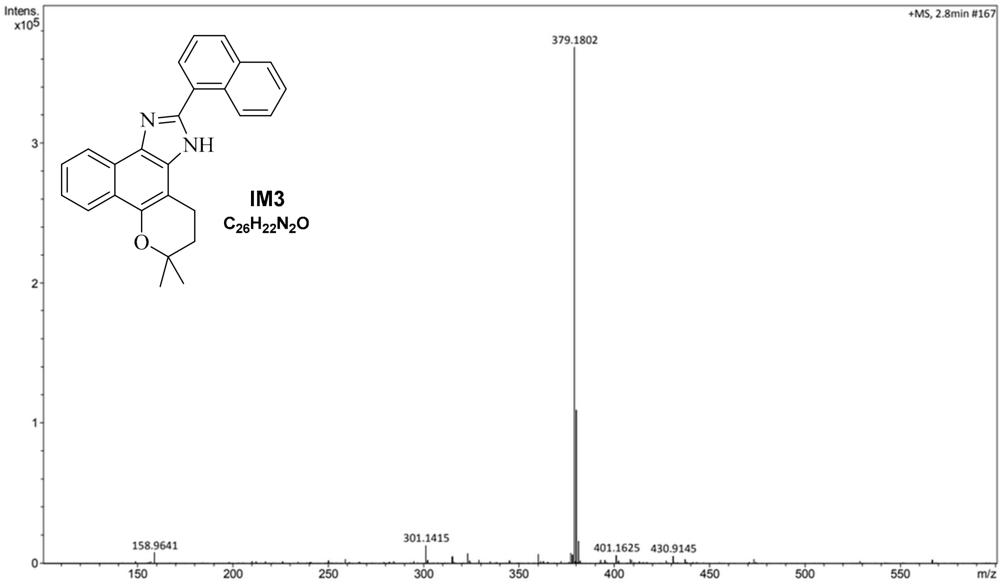

Figure S3. NMR spectra of 4,5-dihydro-6,6-dimethyl-6*H*-2-(naphthalenyl)-pyran[*b*-4,3]naphth[1,2-*d*]imidazole (**IM3)** in DMSO-d_6 and ESI-MS.

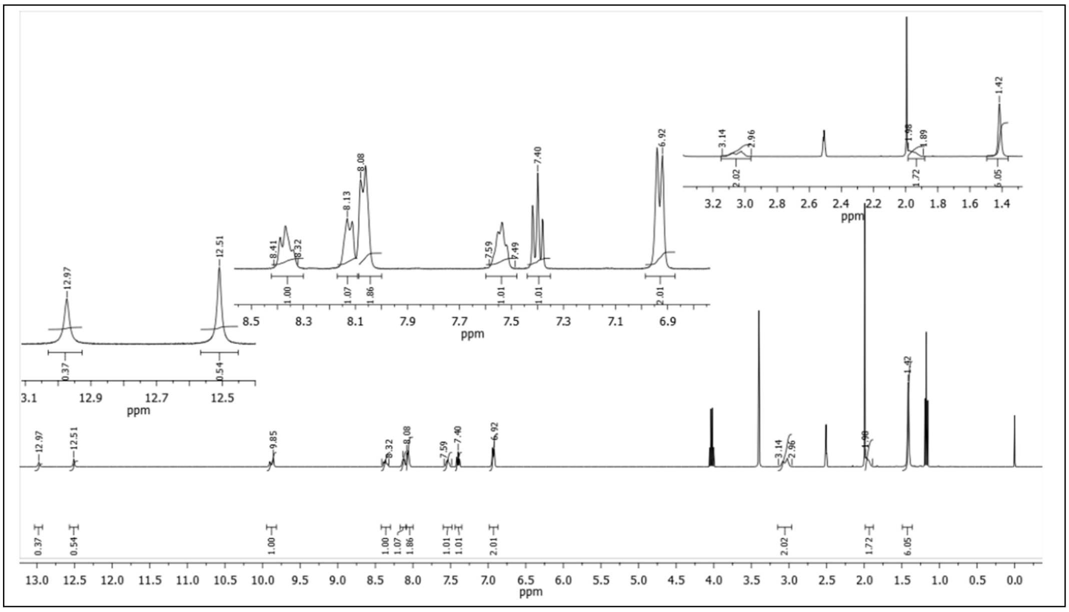
12.97
12.51
0.37
0.54
ppm
8.41
8.32
8.13
8.08
7.59
7.49
7.40
6.92
1.00
1.07
1.86
1.01
1.01
2.01
ppm
3.14
2.96
1.98
1.89
1.42
2.02
1.72
6.05
ppm
9.85
1.00
ppm

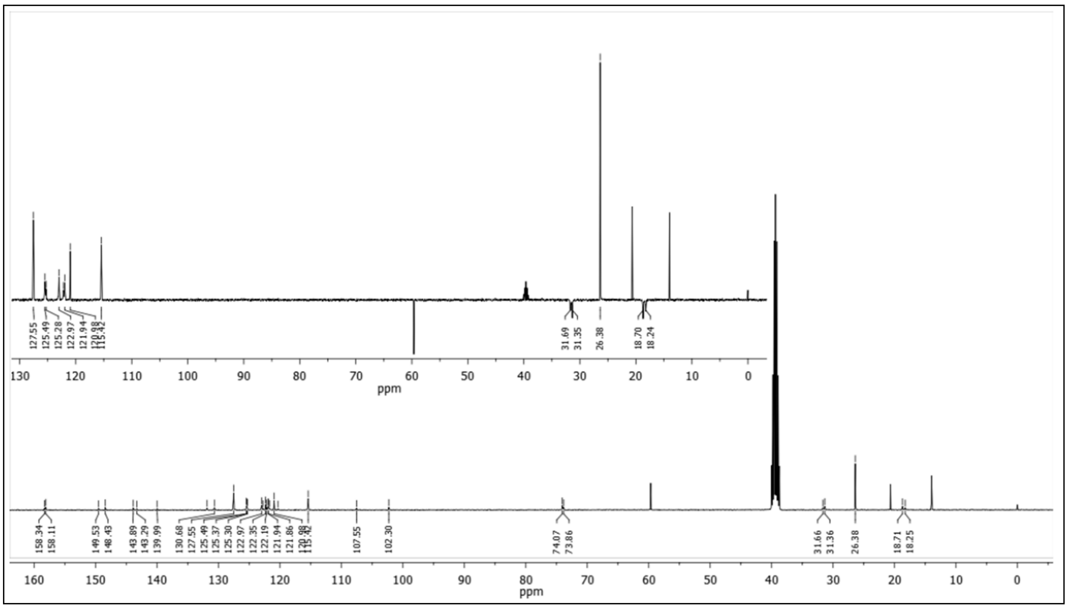
127.55
125.49
125.28
122.97
121.94
31.69
31.35
26.38
18.70
18.24
ppm
158.34
158.11
149.53
148.43
143.89
143.29
139.99
130.68
127.55
125.49
125.37
125.30
122.97
122.35
122.19
121.94
121.86
107.55
102.30
74.07
73.86
31.66
31.36
26.38
18.71
18.25
ppm

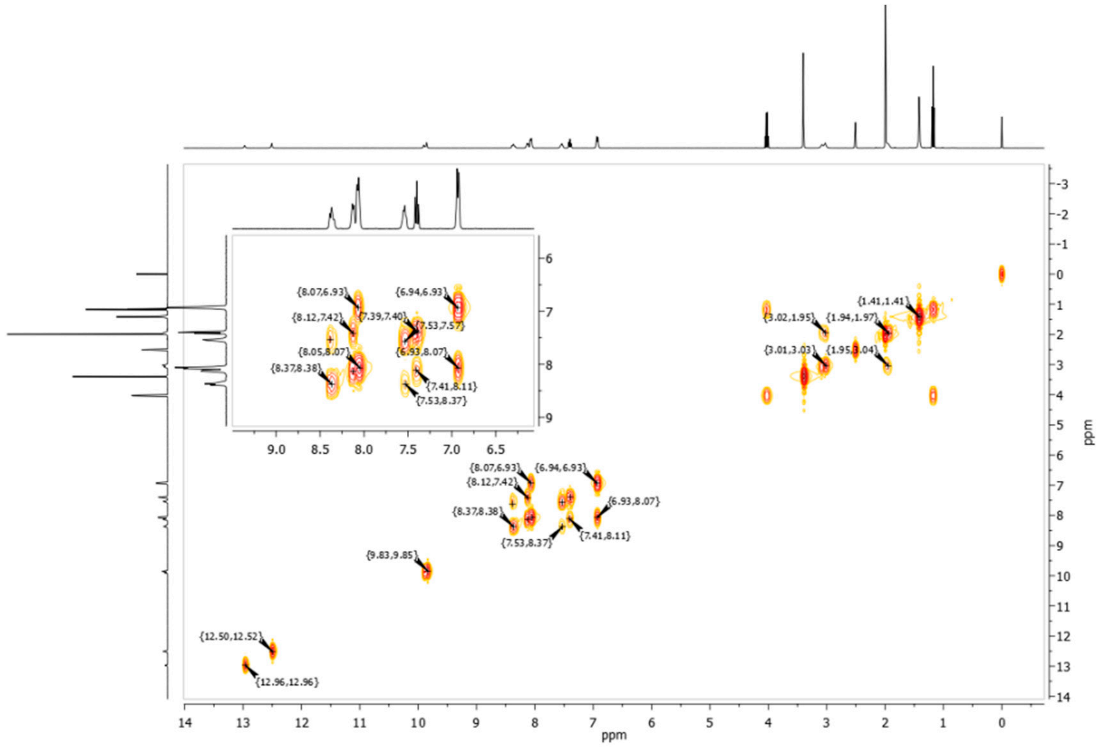
{8.07,6.93}
{6.94,6.93}
{8.12,7.42}
{7.39,7.40}
{7.53,7.57}
{8.05,8.07}
{6.93,8.07}
{8.37,8.38}
{7.41,8.11}
{7.53,8.37}
{3.02,1.95}
{1.94,1.97}
{1.41,1.41}
{3.01,3.03}
{1.95,3.04}
{8.07,6.93}
{6.94,6.93}
{8.12,7.42}
{6.93,8.07}
{8.37,8.38}
{7.53,8.37}
{7.41,8.11}
{9.83,9.85}
{12.50,12.52}
{12.96,12.96}
ppm
ppm

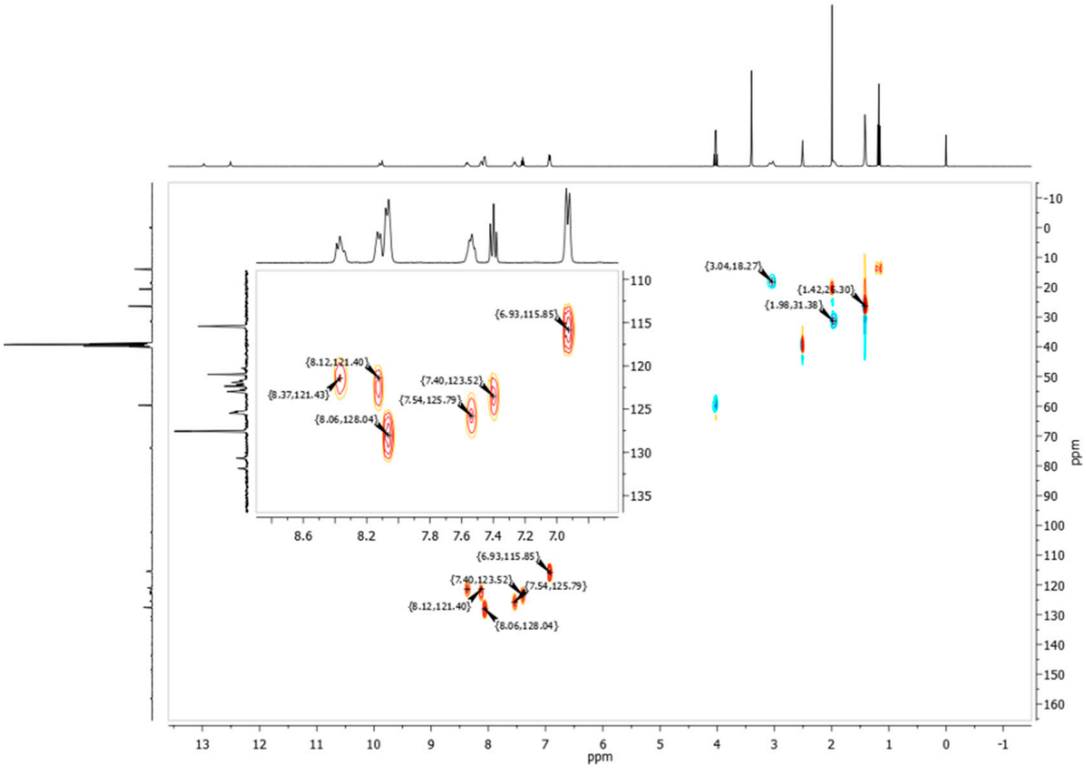
{3.04,18.27}
{1.42,26.30}
{1.98,31.38}
{6.93,115.85}
{8.12,121.40}
{8.37,121.43}
{7.40,123.52}
{7.54,125.79}
{8.06,128.04}
{6.93,115.85}
{7.40,123.52}
{7.54,125.79}
{8.12,121.40}
{8.06,128.04}
ppm
ppm

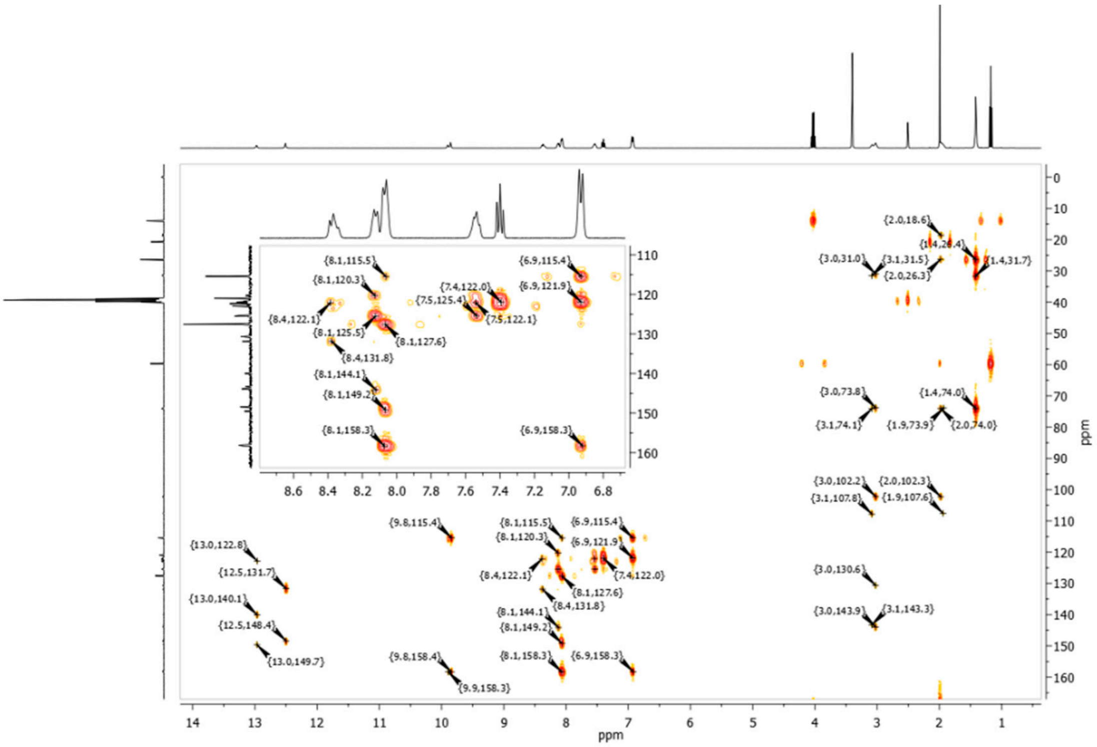

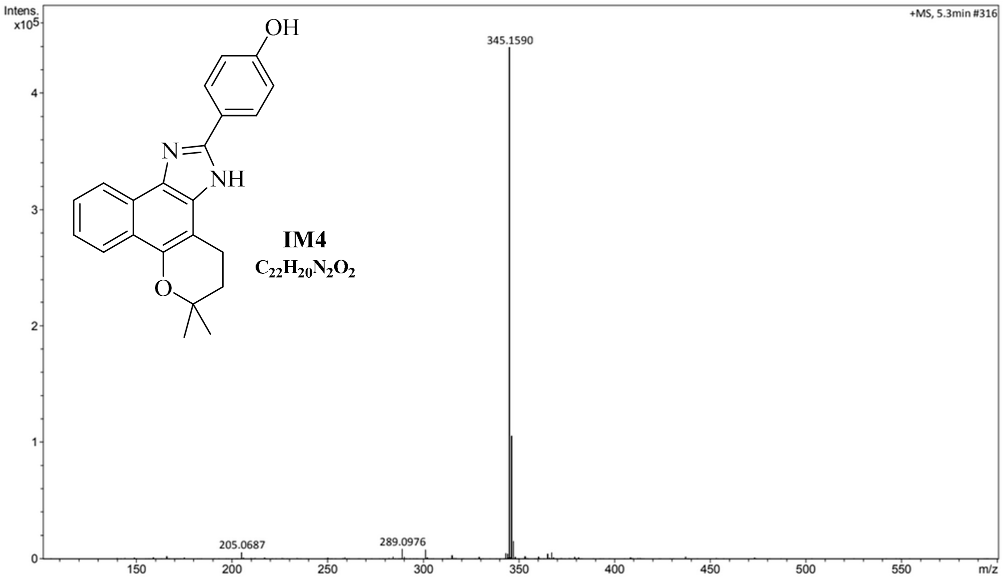

Figure S4. NMR spectra of 4,5-dihydro-6,6-dimethyl-6*H*-2-(4-hidroxyphenyl)-pyran[*b*-4,3]naphth[1,2-*d*]imidazole (**IM4**) in DMSO-d_6 and ESI-MS.

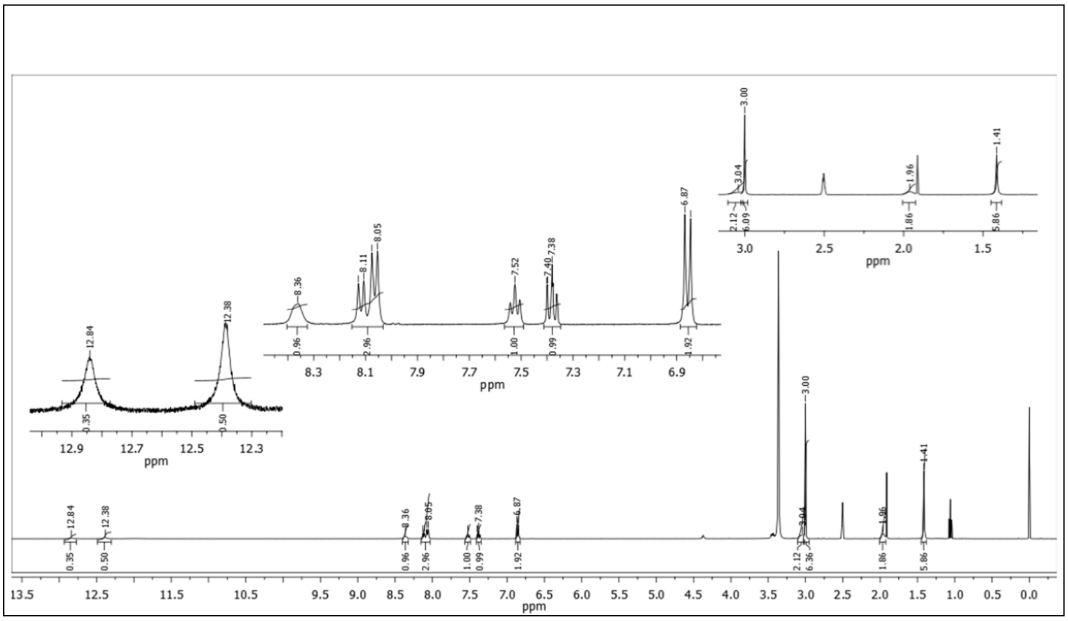

12.84
12.38
0.35
0.50
12.9 12.7 12.5 12.3
ppm
8.36
8.11
8.05
7.52
7.40
7.38
6.87
0.96
2.96
1.00
0.99
1.92
8.3 8.1 7.9 7.7 7.5 7.3 7.1 6.9
ppm
3.00
3.04
1.96
1.41
2.12
6.09
1.86
5.86
3.0 2.5 2.0 1.5
ppm
6.36
13.5 12.5 11.5 10.5 9.5 9.0 8.5 8.0 7.5 7.0 6.5 6.0 5.5 5.0 4.5 4.0 3.5 3.0 2.5 2.0 1.5 1.0 0.5 0.0
ppm

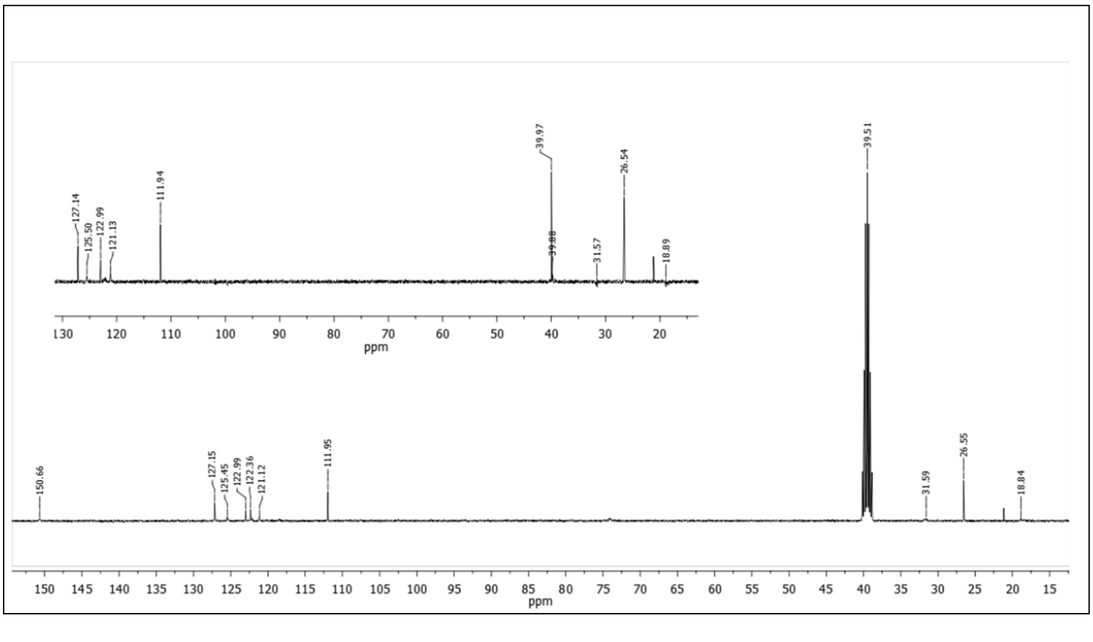

127.14
125.50
122.99
121.13
111.94
39.97
39.88
31.57
26.54
18.89
130 120 110 100 90 80 70 60 50 40 30 20
ppm
39.51
150.66
127.15
125.45
122.99
122.36
121.12
111.95
31.59
26.55
18.84
150 145 140 135 130 125 120 115 110 105 100 95 90 85 80 75 70 65 60 55 50 45 40 35 30 25 20 15
ppm

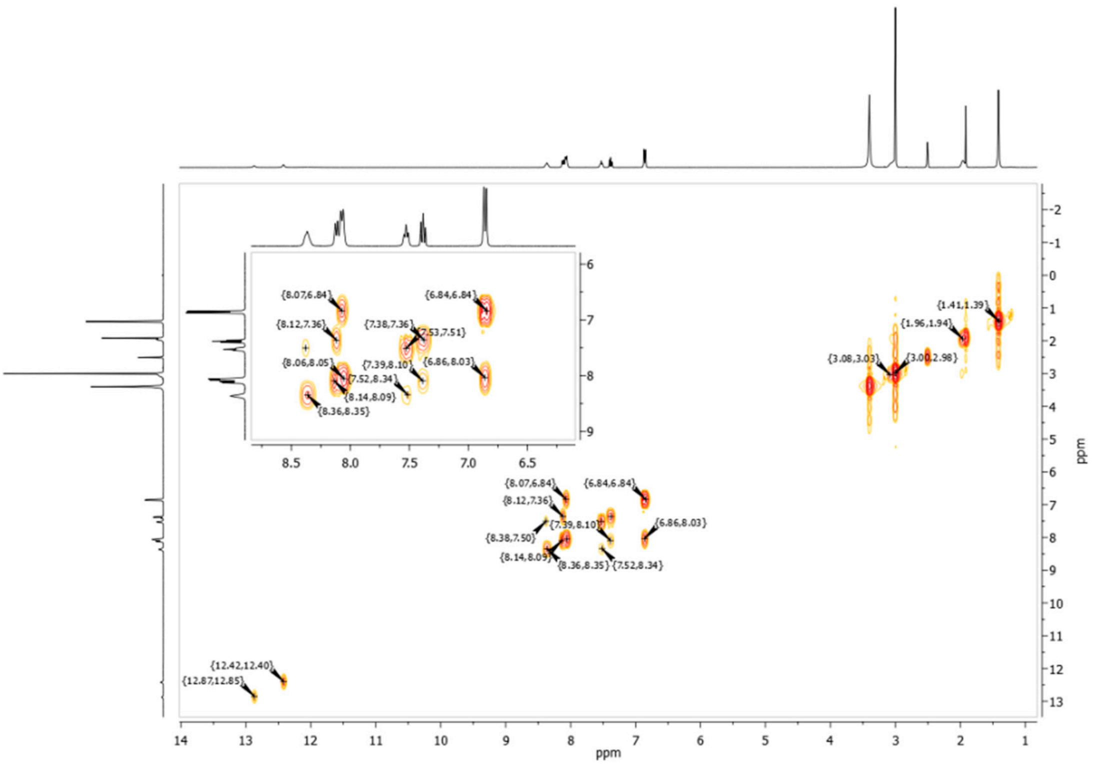

{8.07,6.84}
{6.84,6.84}
{8.12,7.36}
{7.38,7.36}
{7.53,7.51}
{8.06,8.05}
{7.39,8.10}
{6.86,8.03}
{7.52,8.34}
{8.14,8.09}
{8.36,8.35}
{8.38,7.50}
{1.41,1.39}
{1.96,1.94}
{3.08,3.03}
{3.00,2.98}
{12.42,12.40}
{12.87,12.85}
ppm

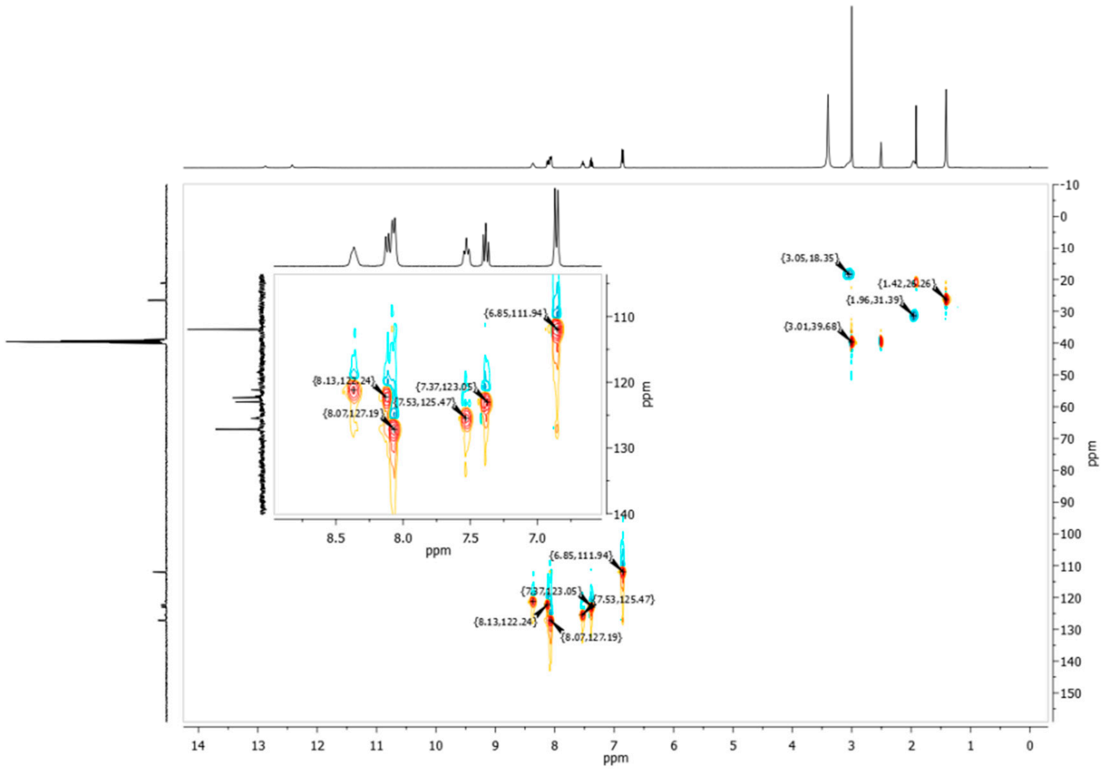

{3.05,18.35}
{1.42,26.26}
{1.96,31.39}
{3.01,39.68}
{6.85,111.94}
{8.13,122.24}
{7.37,123.05}
{7.53,125.47}
{8.07,127.19}
ppm

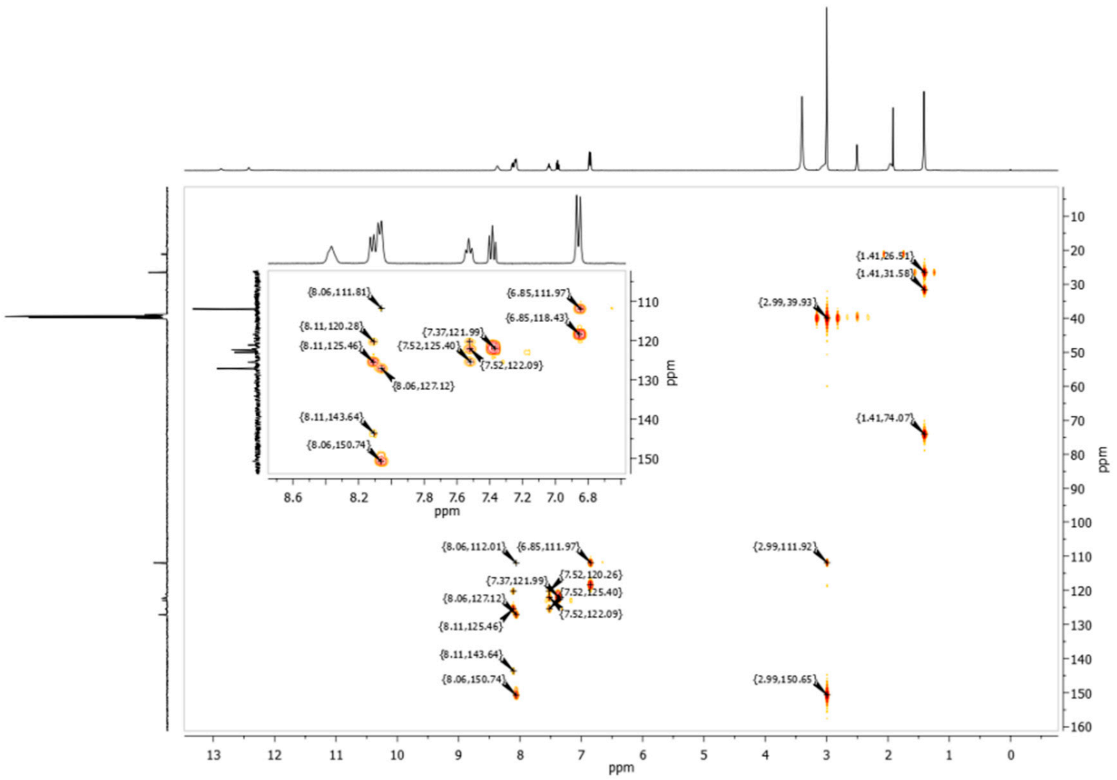

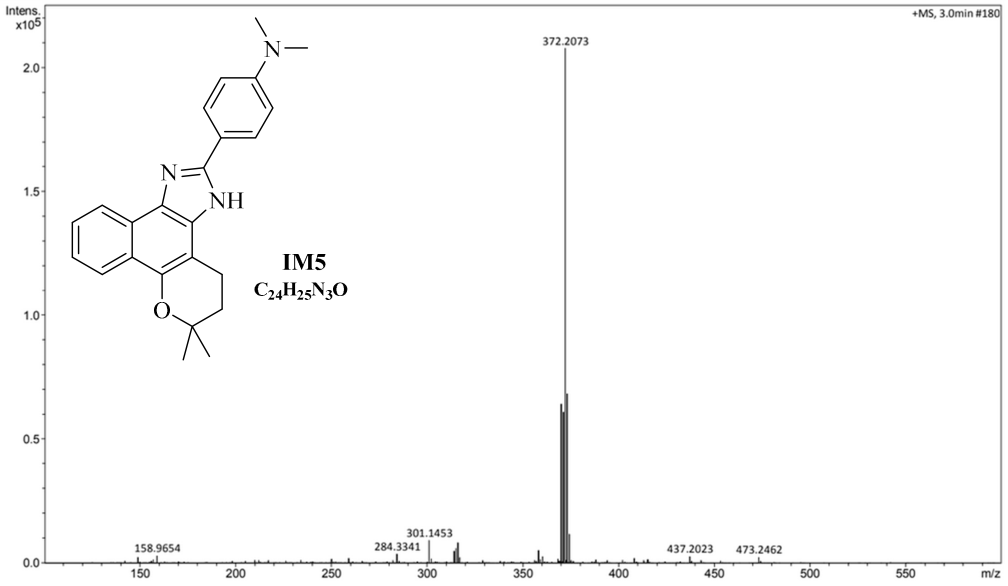

Figure S5. NMR spectra of 4,5-dihydro-6,6-dimethyl-6*H*-2-(4-dimethylaminophenyl)-pyran[*b*-4,3]naphth[1,2-*d*]imidazole **(IM5)** in DMSO-d_6 and ESI-MS.

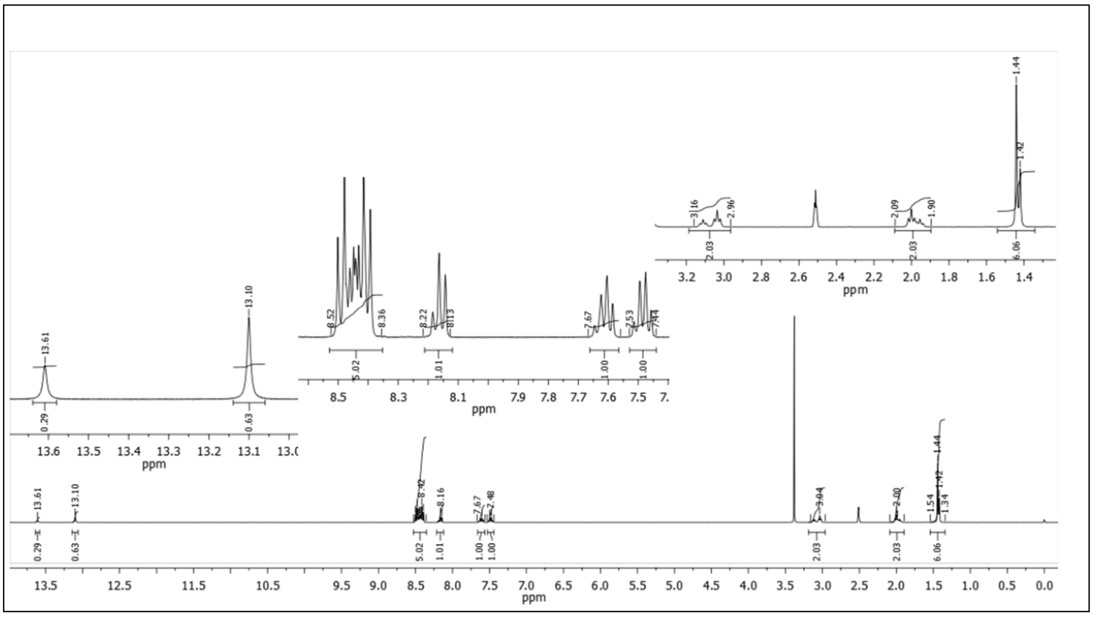
13.61
13.10
0.29
0.63
13.6 13.5 13.4 13.3 13.2 13.1 13.0
ppm
8.52
8.36
8.22
8.13
7.67
7.53
7.44
5.02
1.01
1.00
1.00
8.5 8.3 8.1 7.9 7.8 7.7 7.6 7.5
ppm
3.16
2.96
2.09
1.90
2.03
2.03
1.44
1.42
6.06
3.2 3.0 2.8 2.6 2.4 2.2 2.0 1.8 1.6 1.4
ppm
8.42
8.16
7.67
7.48
3.04
2.00
1.54
1.44
1.42
1.34
13.5 12.5 11.5 10.5 9.5 9.0 8.5 8.0 7.5 7.0 6.5 6.0 5.5 5.0 4.5 4.0 3.5 3.0 2.5 2.0 1.5 1.0 0.5 0.0
ppm

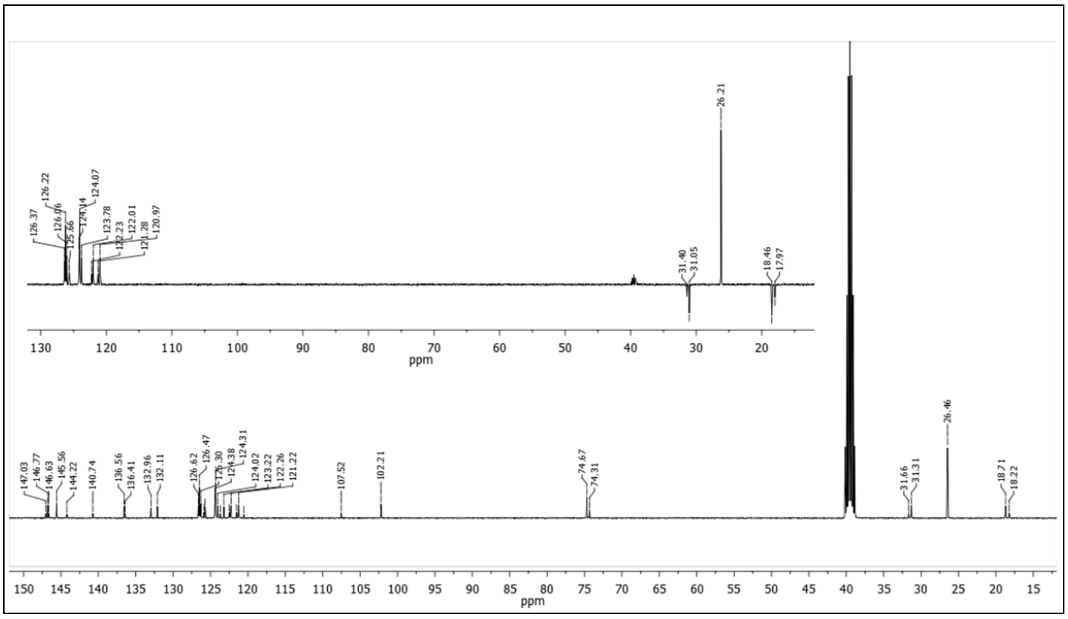
126.37
126.22
126.06
125.66
124.14
124.07
123.78
122.23
122.01
121.28
120.97
26.21
31.40
31.05
18.46
17.97
130 120 110 100 90 80 70 60 50 40 30 20
ppm
147.03
146.77
146.63
145.56
144.22
140.74
136.56
136.41
132.96
132.11
126.62
126.47
126.30
124.38
124.31
124.02
123.22
122.26
121.22
107.52
102.21
74.67
74.31
31.66
31.31
26.46
18.71
18.22
150 145 140 135 130 125 120 115 110 105 100 95 90 85 80 75 70 65 60 55 50 45 40 35 30 25 20 15
ppm

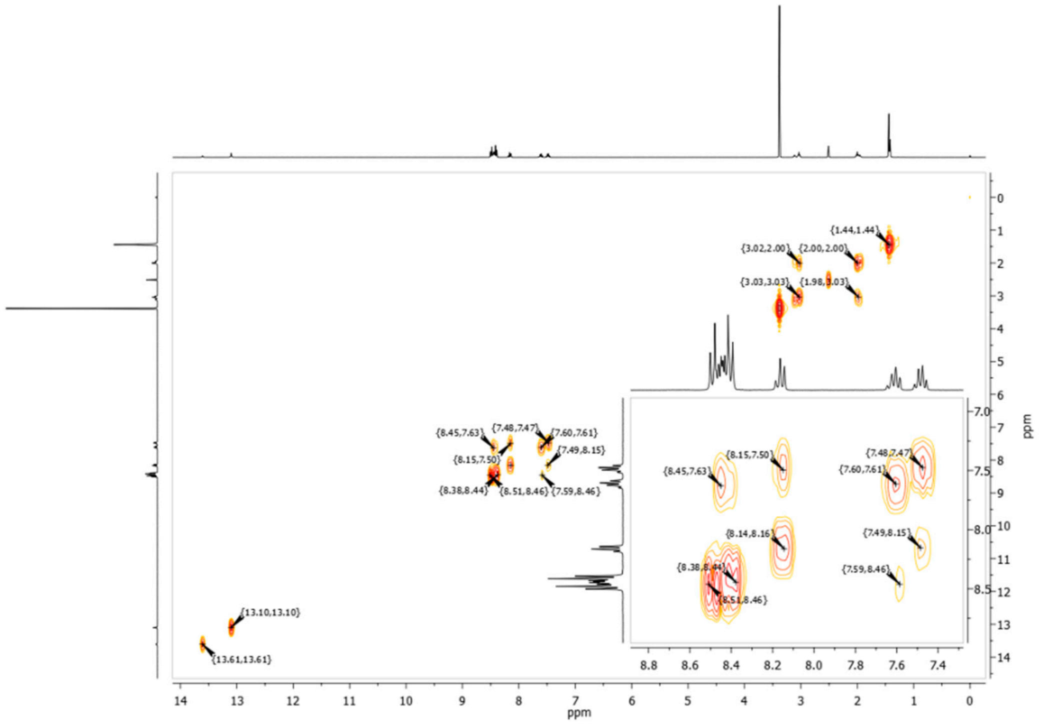

{1.44,1.44}
{3.02,2.00}
{2.00,2.00}
{3.03,3.03}
{1.98,3.03}
{8.45,7.63}
{7.48,7.47}
{7.60,7.61}
{8.15,7.50}
{7.49,8.15}
{8.38,8.44}
{8.51,8.46}
{7.59,8.46}
{8.14,8.16}
{13.10,13.10}
{13.61,13.61}
ppm

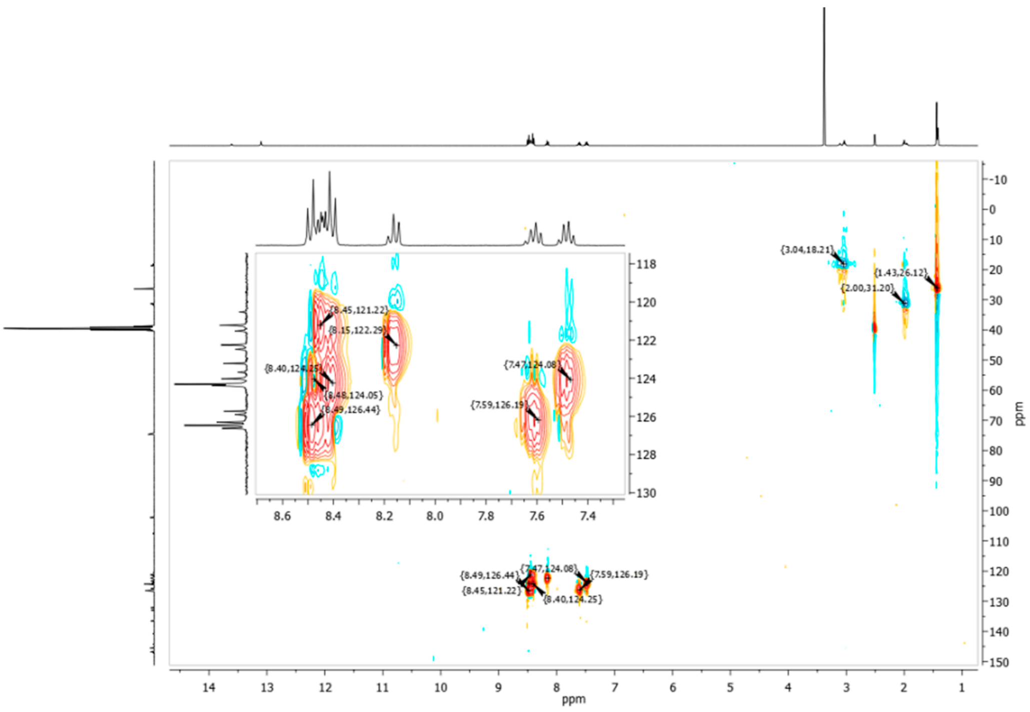

{3.04,18.21}
{1.43,26.12}
{2.00,31.20}
{8.45,121.22}
{8.15,122.29}
{7.47,124.08}
{8.40,124.25}
{8.48,124.05}
{7.59,126.19}
{8.49,126.44}
ppm

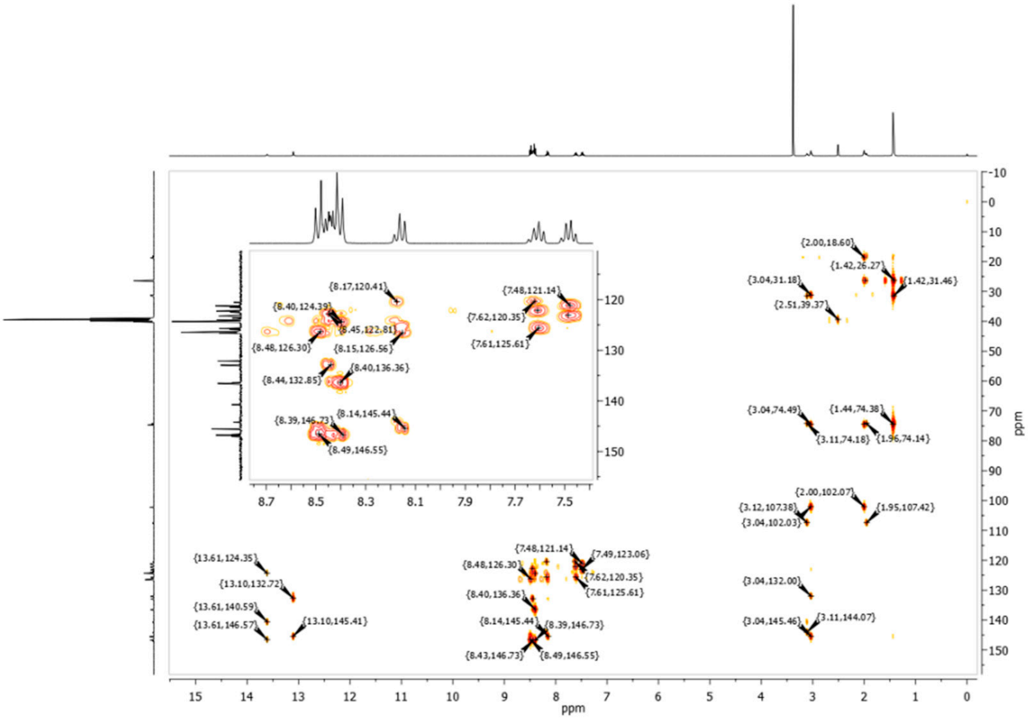

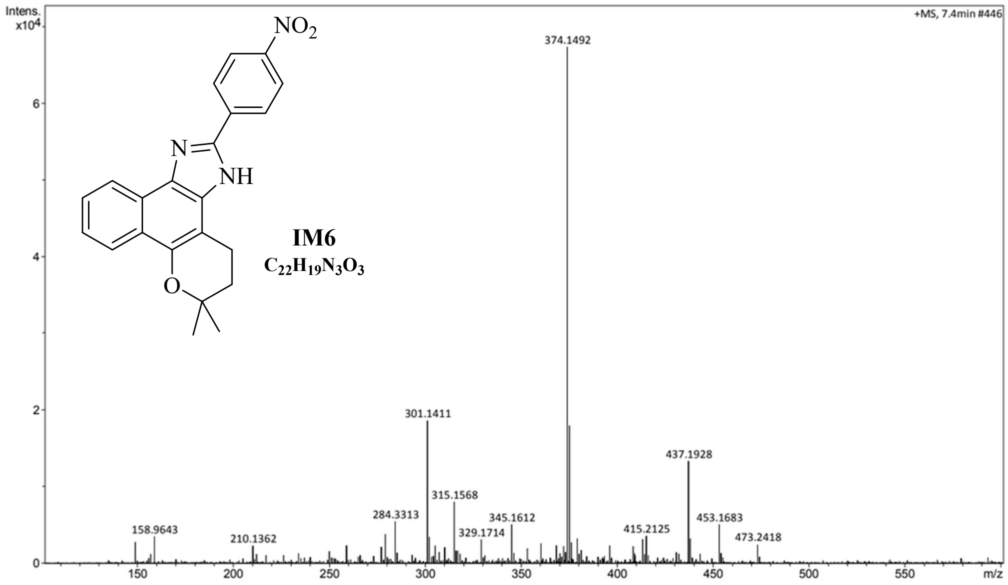

Figure S6. NMR spectra of 4,5-dihydro-6,6-dimethyl-6*H*-2-(4-nitrophenyl)-pyran[*b*-4,3]naphth[1,2-*d*]imidazole (**IM6**) in DMSO-d_6 and ESI-MS.

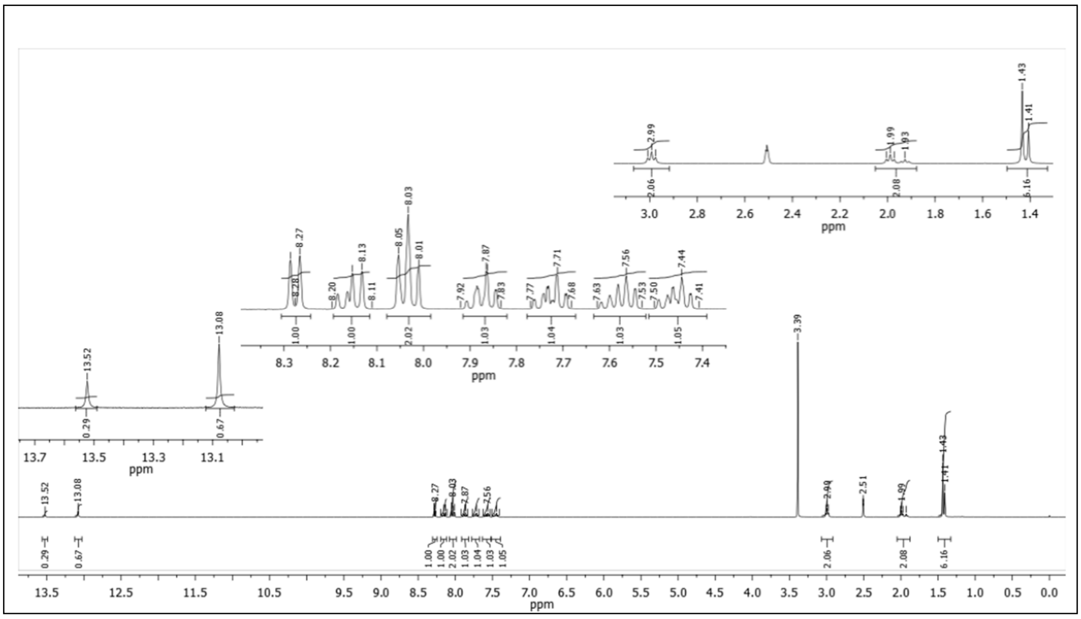

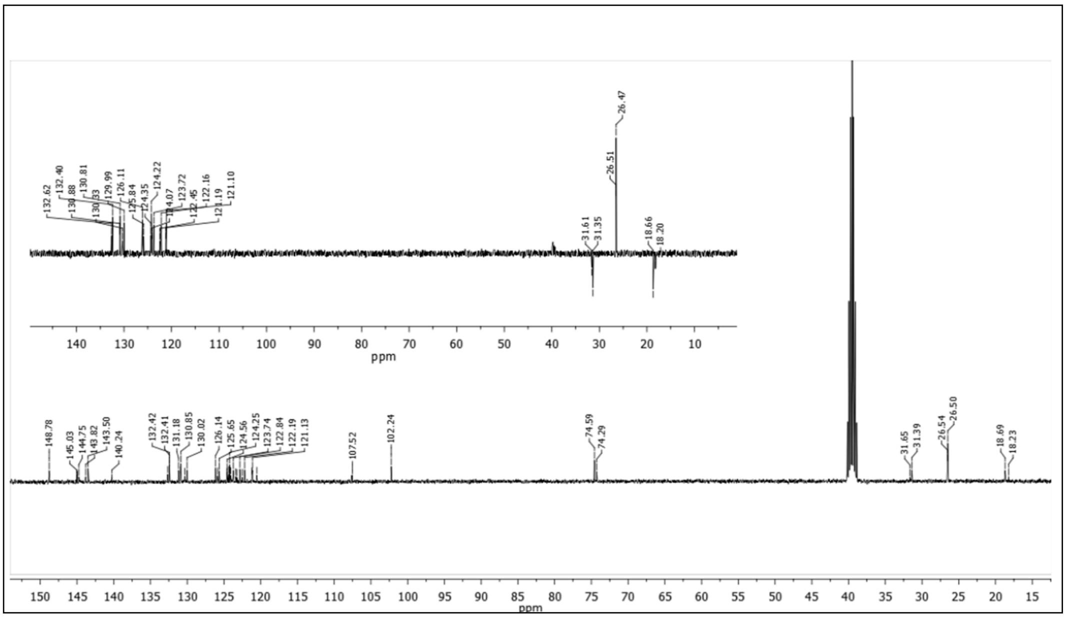

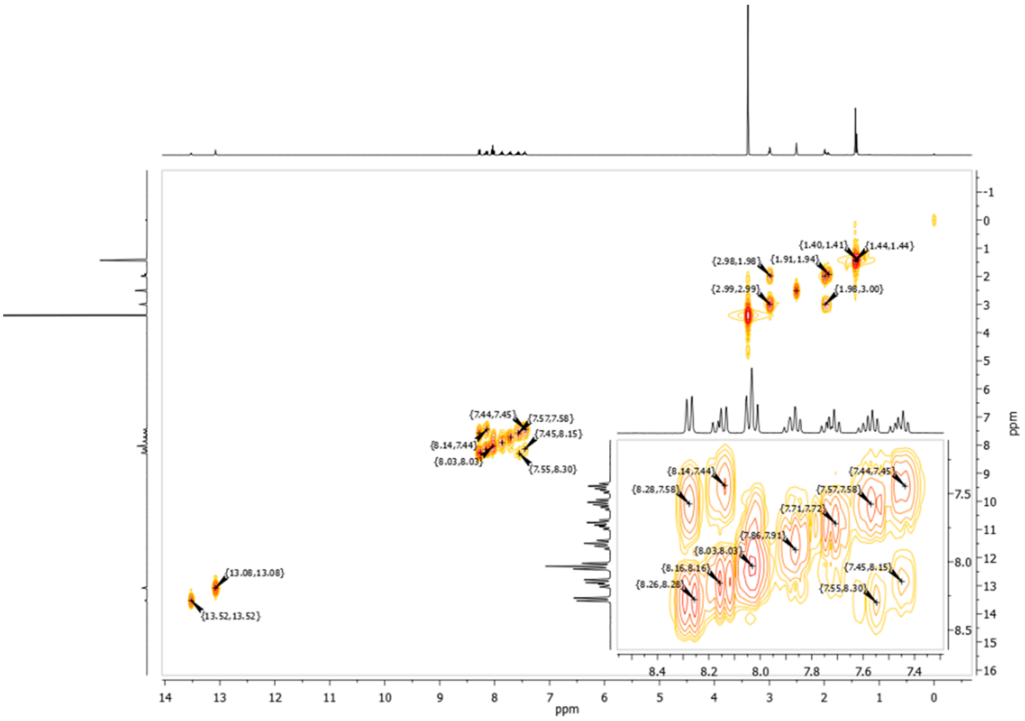
{2.98,1.98}
{1.91,1.94}
{1.40,1.41}
{1.44,1.44}
{2.99,2.99}
{1.98,3.00}
{7.44,7.45}
{7.57,7.58}
{7.45,8.15}
{8.14,7.44}
{8.03,8.03}
{7.55,8.30}
{13.08,13.08}
{13.52,13.52}
{8.14,7.44}
{8.28,7.58}
{7.44,7.45}
{7.57,7.58}
{7.71,7.72}
{7.86,7.91}
{8.03,8.03}
{8.16,8.16}
{8.26,8.28}
{7.45,8.15}
{7.55,8.30}
ppm
ppm

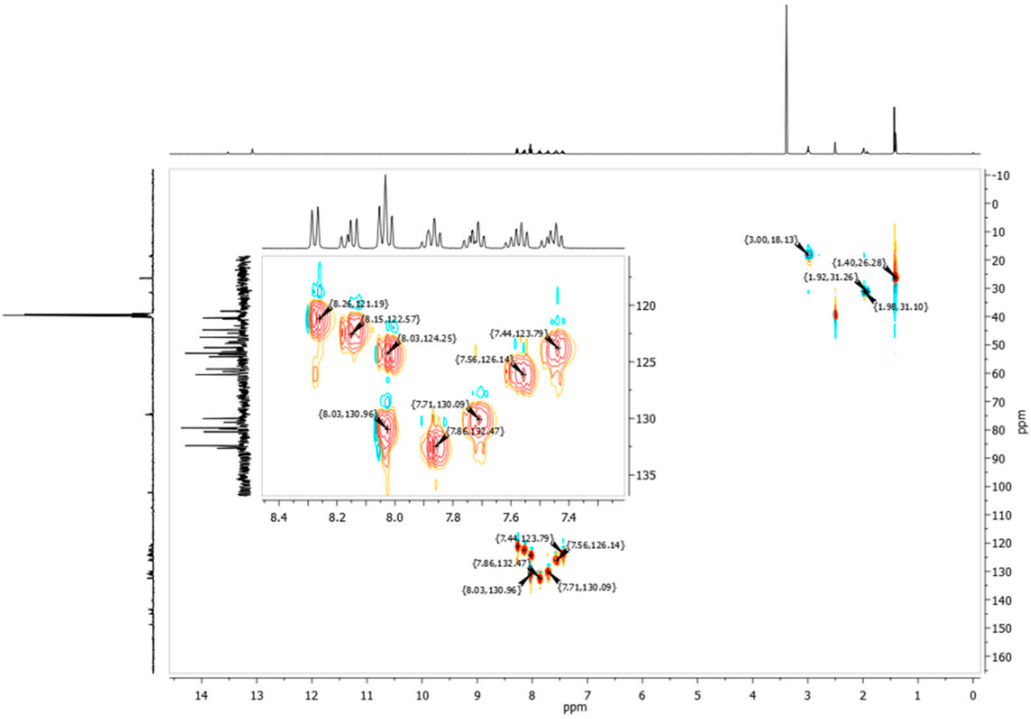
{3.00,18.13}
{1.40,26.28}
{1.92,31.26}
{1.98,31.10}
{8.26,121.19}
{8.15,122.57}
{8.03,124.25}
{7.44,123.79}
{7.56,126.14}
{8.03,130.96}
{7.71,130.09}
{7.86,132.47}
{7.44,123.79}
{7.56,126.14}
{7.86,132.47}
{8.03,130.96}
{7.71,130.09}
ppm
ppm

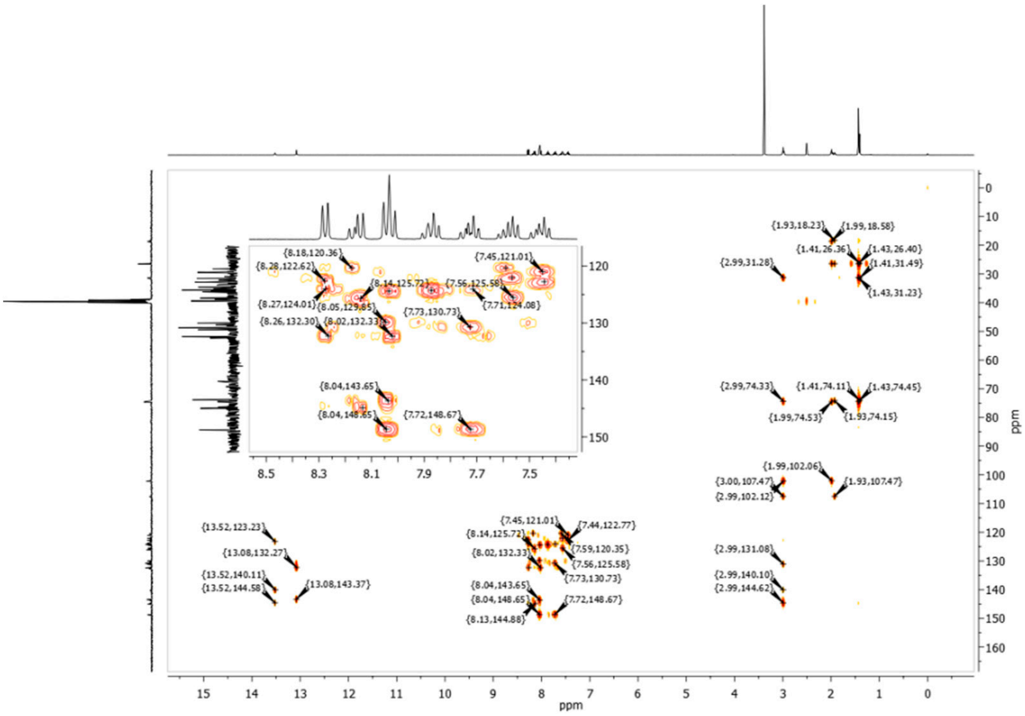

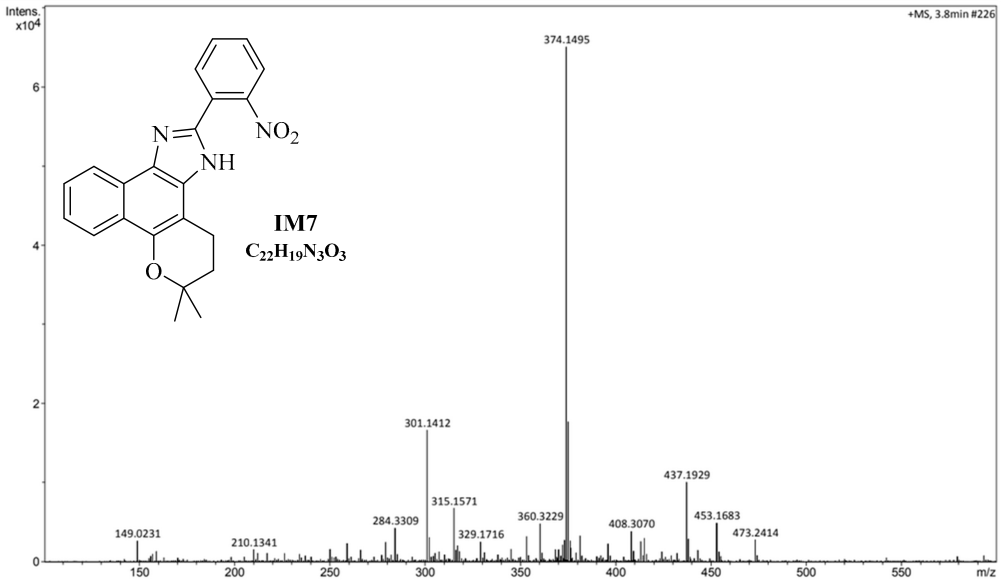

Figure S7. NMR spectra of 4,5-dihydro-6,6-dimethyl-6*H*-2-(2-nitrophenyl)-pyran[*b*-4,3]naphth[1,2-*d*]imidazole (**IM7**) in DMSO-d_6 and ESI-MS.

2. Solvatochromism study

Table S1. Wavelength (nm) and absorbance of the scanning spectra of the naphth[1,2-*d*]imidazoles obtained in the solvatochromism study.

SOLVATOCHROMISM STUDY λ_{Abs} (nm) and absorbance				
**COMPOUND**	**HEXANE**	**CH_2Cl_2**	**DMSO**	**CH_3OH**
**IM1**	313 (0.088)<br>328 (0.068)	315 (0.107)<br>329 (0.117)	320 (0.111)<br>333 (0.102)	314 (0.068)<br>328 (0.077)
**IM2**	291 (0.228)<br>344 (0.203)	289 (0.333)<br>348 (0.330)	360 (0.125)<br>450 (0.149)	288 (0.355)<br>343 (0.375)
**IM3**	348 (0.152)	306 (0.209)<br>355 (0.334)	305 (0.189)<br>360 (0.313)	288 (0.243)<br>343 (0.320)
**IM4**	Not observed	360 (0.125)<br>450 (0.149)	346 (0.315)<br>362 (0.306)	342 (0.342)<br>352 (0.293)
**IM5**	302 (0.155)<br>352 (0.130)	321 (0.271)<br>374 (0.426)<br>485 (0.072)	307 (0.494)<br>357 (0.614)<br>375 (0.573)	300 (0.429)<br>353 (0.530)
**IM6**	290 (0.083)<br>413 (0.234	321 (0.096)<br>428 (0.226)	315 (0.098)<br>433 (0.235)	298 (0.088)<br>414 (0.234)
**IM7**	294 (0.235)<br>341 (0.241)<br>427 (0.088)	287 (0.220)<br>336 (0.238)	281 (0.256)<br>341 (0.229)	333 (0.184)

3. Absorbance and fluorescence emission spectrum

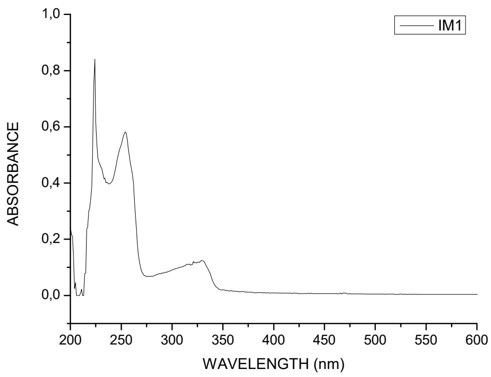

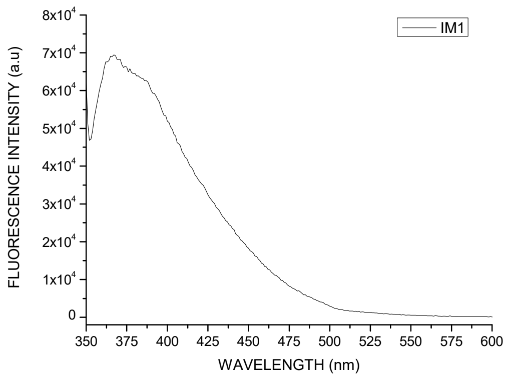

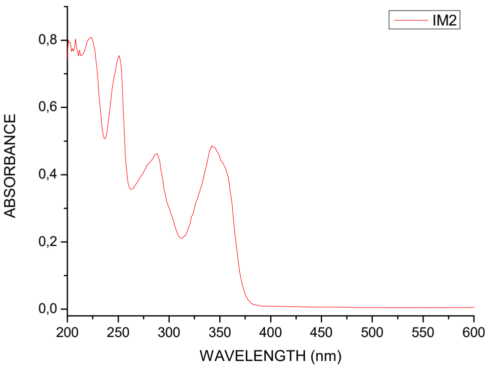
IM2
ABSORBANCE
0,0
0,2
0,4
0,6
0,8
200 250 300 350 400 450 500 550 600
WAVELENGTH (nm)

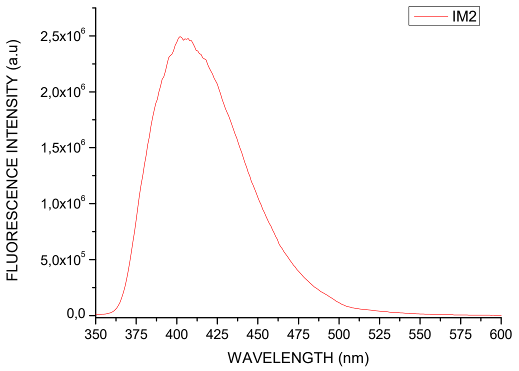
IM2
FLUORESCENCE INTENSITY (a.u)
0,0
$5,0x10^5$
$1,0x10^6$
$1,5x10^6$
$2,0x10^6$
$2,5x10^6$
350 375 400 425 450 475 500 525 550 575 600
WAVELENGTH (nm)

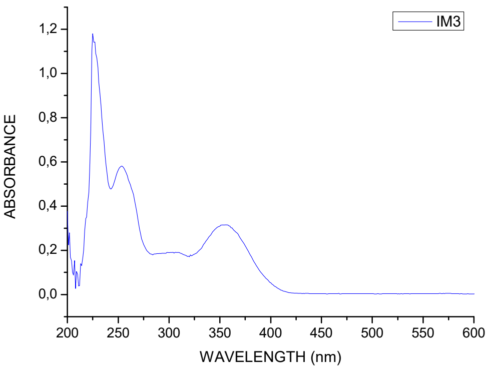
IM3
ABSORBANCE
0,0
0,2
0,4
0,6
0,8
1,0
1,2
200 250 300 350 400 450 500 550 600
WAVELENGTH (nm)

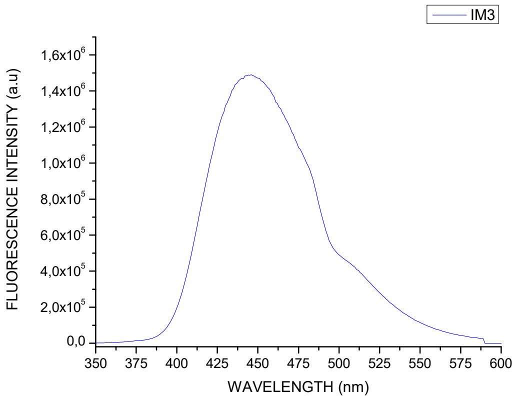
IM3
FLUORESCENCE INTENSITY (a.u)
0,0
$2,0x10^5$
$4,0x10^5$
$6,0x10^5$
$8,0x10^5$
$1,0x10^6$
$1,2x10^6$
$1,4x10^6$
$1,6x10^6$
350 375 400 425 450 475 500 525 550 575 600
WAVELENGTH (nm)

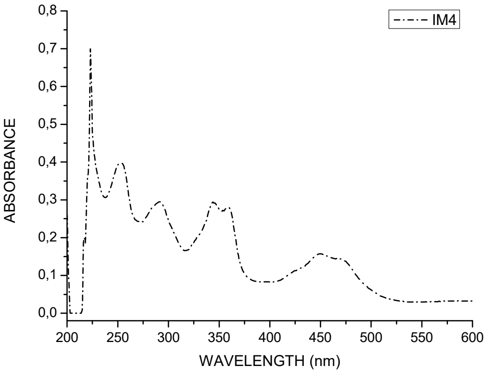
IM4
ABSORBANCE
0,0
0,1
0,2
0,3
0,4
0,5
0,6
0,7
0,8
200 250 300 350 400 450 500 550 600
WAVELENGTH (nm)

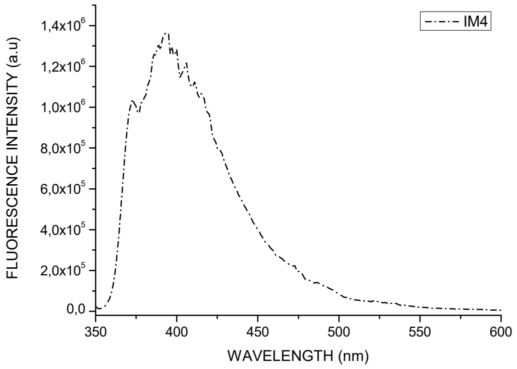
IM4
FLUORESCENCE INTENSITY (a.u)
0,0
$2,0x10^5$
$4,0x10^5$
$6,0x10^5$
$8,0x10^5$
$1,0x10^6$
$1,2x10^6$
$1,4x10^6$
350 400 450 500 550 600
WAVELENGTH (nm)

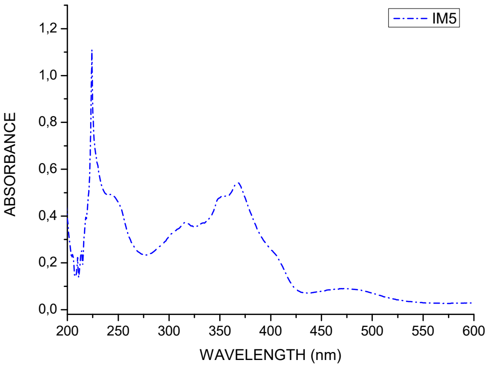
IM5
ABSORBANCE
0,0
0,2
0,4
0,6
0,8
1,0
1,2
200 250 300 350 400 450 500 550 600
WAVELENGTH (nm)

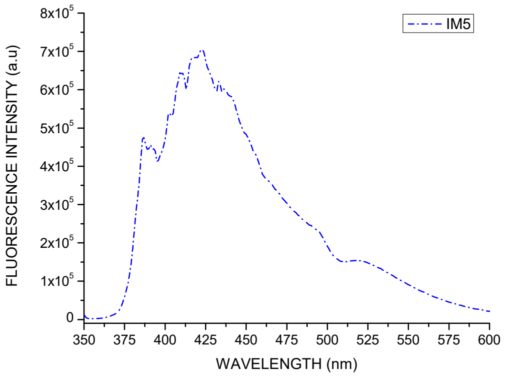
IM5
FLUORESCENCE INTENSITY (a.u)
0
$1x10^5$
$2x10^5$
$3x10^5$
$4x10^5$
$5x10^5$
$6x10^5$
$7x10^5$
$8x10^5$
350 375 400 425 450 475 500 525 550 575 600
WAVELENGTH (nm)

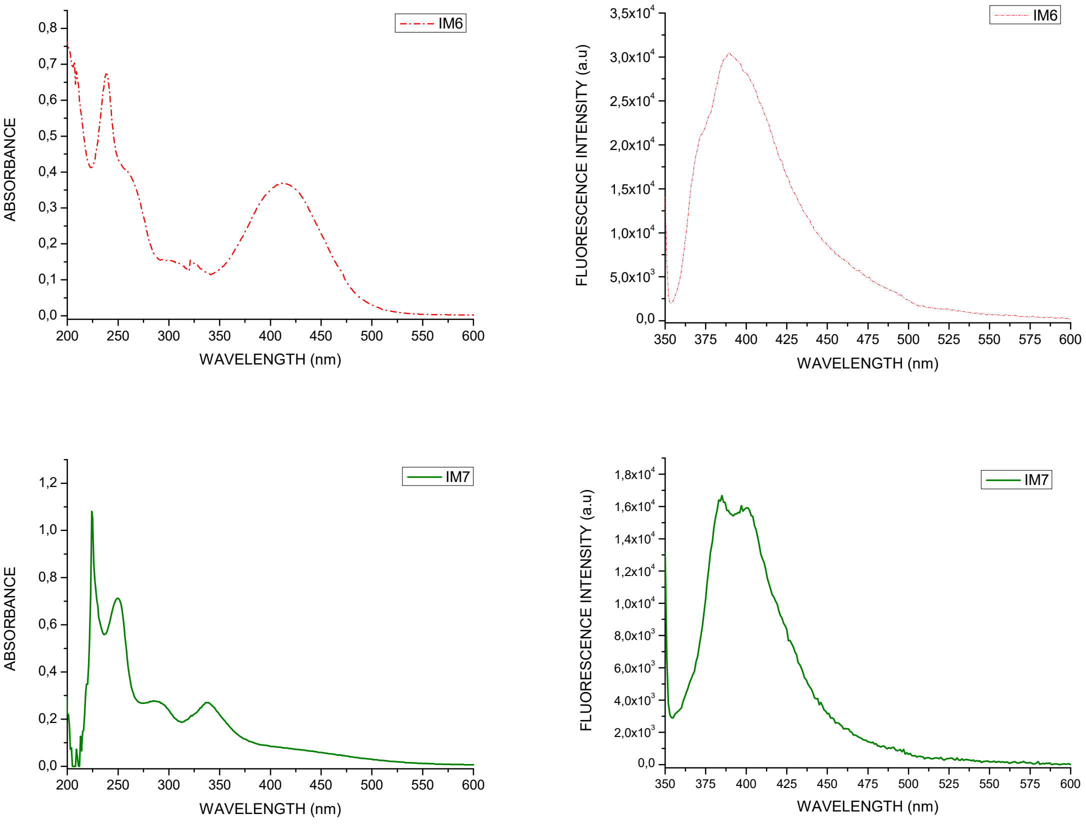

a. Solvents used solvents: IM2 – DMSO; IM6 - CH_3OH; IM1, IM3, IM4, IM5 - CH_2Cl_2; and IM7 hexane.
b. Excitation wavelength of 345 nm for all compounds.

Figure S8. Absorbance and emission spectra of naphth[1,2-*d*]imidazoles (**IM1–IM7)**.
